# Composite Polymers Development and Application for Polymer Electrolyte Membrane Technologies—A Review

**DOI:** 10.3390/molecules25071712

**Published:** 2020-04-08

**Authors:** Gabriele G. Gagliardi, Ahmed Ibrahim, Domenico Borello, Ahmad El-Kharouf

**Affiliations:** 1 Department of Mechanical and Aerospace Engineering, Sapienza Università di Roma, 00184 Rome, Italy; Gabriele.gagliardi@uniroma1.it; 2 School of Chemical Engineering, University of Birmingham, Birmingham B15 2TT, UK; AXI763@student.bham.ac.uk

**Keywords:** composite membranes, electrolyte, PEM, fuel cells, electrolysers

## Abstract

Nafion membranes are still the dominating material used in the polymer electrolyte membrane (PEM) technologies. They are widely used in several applications thanks to their excellent properties: high proton conductivity and high chemical stability in both oxidation and reduction environment. However, they have several technical challenges: reactants permeability, which results in reduced performance, dependence on water content to perform preventing the operation at higher temperatures or low humidity levels, and chemical degradation. This paper reviews novel composite membranes that have been developed for PEM applications, including direct methanol fuel cells (DMFCs), hydrogen PEM fuel cells (PEMFCs), and water electrolysers (PEMWEs), aiming at overcoming the drawbacks of the commercial Nafion membranes. It provides a broad overview of the Nafion-based membranes, with organic and inorganic fillers, and non-fluorinated membranes available in the literature for which various main properties (proton conductivity, crossover, maximum power density, and thermal stability) are reported. The studies on composite membranes demonstrate that they are suitable for PEM applications and can potentially compete with Nafion membranes in terms of performance and lifetime.

## 1. Background

During the last 100 years the world average temperature has increased by almost 0.8 °C [1], becoming the most critical environmental issue of our time. Even though there are many different factors responsible, the greatest concern is greenhouse gas emissions due to human activities linked to energy production and use. In this sense, governments worldwide are acting to take measures to revise their energy mix by reducing fossil fuels usage and promoting alternative sources. The European Union, with the objectives set in the 20-20-20 pack, put forward strict targets to be reached before 2020, namely 20% reduction of greenhouse gases, 20% primary energy production from renewables, and 20% of biofuels burned in transportation. Moreover, recently a medium-long term strategy was agreed, stating that the European energy efficiency should be improved by 27% and the renewables energy input should increase by up to the 27% of the total share before 2030. Within this overall framework, it is becoming increasingly important that research and development of new technologies are intensified to allow the penetration of more efficient energy conversion systems. In this context, polymer electrolyte membrane technologies can play an important role.

### 1.1. Polymer Electrolyte Membrane Technologies

Fuel cells and elecrolysers are energy conversion systems that electrochemically convert energy from chemical (stored in a fuel) to electric and vice versa without any intermediated combustion process. This offers superior efficiency and performance compared to the incumbent combustion-based energy generation technologies [2,3,4,5]. Fuel cells are eco-friendly devices with potential zero emission at the point of use. Moreover, if the so-called energy vectors, used in fuel cells, were generated by thermochemical processes from biomass or from electrochemical processes utilising renewable electric energy sources, the resulting carbon dioxide cycle would be null. So, they are considered to be the energy conversion devices of the future. In addition, they are a silent technology, without noise or vibration, and their design flexibility allows for simple construction and a diverse range of applications including portable, stationary, and transportation.

In general, electrochemical devices, including fuel cells and electrolysers, consist of two electrodes—anode and cathode—separated by an electrolyte with the purpose to allow the passage of ions generated during the redox half reactions. At the anode side, the oxidation reaction takes place while the reduction reaction occurs at the cathode side. The electrolyte conducts the produced/required ions to complete the reactions at the electrodes and serves as a separator between the anode and cathode reactants in the fuel cell and electrolyser technologies.

This study will focus on Proton Exchange Membrane (PEM) technologies, namely, Hydrogen Polymer Electrolyte Membrane Fuel Cells (H_2_ PEMFCs), Direct Methanol Fuel Cells (DMFCs) and Polymer Electrolyte Membrane Water Electrolysers (PEMWEs). PEM technologies fall under the low temperature fuel cells category with operating temperatures up to 90 °C. Protons (hydrogen ions) are the transported ion through the PEM structure. Below, the thermodynamic, state of art and technical challenges of the three technologies are briefly described.

#### 1.1.1. H_2_ PEMFC

PEMFCs utilize hydrogen as a fuel and exploit the electrochemical reaction of hydrogen and oxygen to produce electrical energy. Protons pass through the membrane reaching the cathode while electrons are forced to flow through an external circuit. Protons, electrons, and oxygen react at the cathode producing water. Reactions involved in the chemical process are described below:

(1) $$\mathrm{At}\;\mathrm{the}\;\mathrm{anode}:\;H_{2}\to 2H^{+}+2e^{-}$$

(2) At the cathode: 12O2+2H++2e−→H2O

(3) Overall reaction: H2+12O2→H2O

PEMFC advantages include high efficiency, fast response to load, high power density and low operating temperature [6,7]. However, PEMFCs are expensive due to the use of expensive catalyst materials, have durability issues and are challenging for mass production [8,9,10]. PEMFCs applications focus on transportation, distributed/stationary and portable power generation: Toyota, Honda, and Hyundai have already introduced their fuel cell electric vehicles (FCEV) to the market.

The durability of the polymer membrane is a crucial factor affecting the lifetime of the stack. Industry requirements for automotive fuel cell stacks durability is 5000 h with a performance drop of no more than 10% [11]. Nafion is the only membrane reported to achieve this requirement. Also, at elevated temperatures (> 90 °C) the durability is further reduced due to the dehydration of the membrane and the subsequent drop in proton conductivity. However, operation at higher temperatures is desirable as it allows for quicker reaction kinetics and simpler water and heat management [12]. This means a smaller fuel cell system can be employed in vehicles to provide the same power output.

#### 1.1.2. DMFC

Methanol can be used in PEM fuel cells as a replacement fuel to Hydrogen. The first physical advantage is the liquid natural state of this compound that facilitates its transport, avoiding delicate compressed gas infrastructure development or heavy metal hydrides. It has higher volumetric energy density than compressed hydrogen at 350bar, has low volatility and is almost environmentally neutral in its degradation [13] [14]. In addition, Methanol can be produced from syngas (carbon monoxide and hydrogen compound), allowing to primarily exploit renewable feedstocks as biomass or solid wastes. In recent years, this idea has attracted a lot of attention to find a possible carbon-neutral energy cycle [15]. DMFCs could find application as alternative power sources for vehicle propulsion [16] but are mainly considered for portable applications [17].

Reactions occur at the two electrodes of a DMFC upon the catalyst active area as shown Equations (4)–(6). Again, the flow of electrons through the external circuit accompanied by the flow of protons through the polymer electrolyte allows the reactions to occur and electrical power to be produced [18].

(4) $$\mathrm{At}\;\mathrm{the}\;\mathrm{anode}:\;{\mathrm{CH}}_{3}\mathrm{OH}+H_{2}O\to 6H^{+}+6e^{-}+{\mathrm{CO}}_{2}$$

(5) At the cathode: 32O2+6H++6e−→3H2O

(6) Overall reaction:CH3OH+32O2→2H2O+CO2

However, DMFCs suffer from low performance due to methanol crossover. Methanol crossover happens when methanol molecules diffuse through the membrane and are directly oxidized by oxygen on the cathode, causing a mixed potential so, consequently, a decrease in cell performance [19]. Although methanol has a high energy density (about 1.8 kWh kg^−1^ or 1.7 kWh L^−1^), it must be diluted in order to reduce methanol crossover. A consequence of dilution is that the cell stack dimensions must be proportionally increased, making it challenging to utilize on small and portable devices.

Moreover, it was found that methanol crossover slows down the rate of reaction at the cathode. It has been reported that the rate of reactions not only can be accelerated by adding more platinum–ruthenium catalyst that has a negative effect on the cost, but also by selecting proper membranes and oxygen tolerant cathodes [20].

Also the carbon dioxide generated during the methanol oxidation can further increase the concentration losses: CO_2_ bubbles can obstruct GDL pores, reducing the available volume aimed to transport methanol towards the anode catalyst [21] causing a decrease in generated power by more than 40% [22], especially at small flow rate and high current density.

#### 1.1.3. Electrolysers

Electrolysis is an electrochemical process where water molecules split into hydrogen and oxygen gases using the supplied direct electric current. In PEMWEs, the reverse of the PEMFCs reactions described above Equations. (1)–(3) take place, see Equations (7)–(9).

(7) At the anode:H2O→2H++12O2+2e−

(8) $$\mathrm{At}\;\mathrm{the}\;\mathrm{cathode}:2H^{+}+2e^{-}\to H_{2}$$

(9) Overall reaction: H2O→H2+12O2

PEMWE is one of the favorable methods for conversion of renewable energy to high purity hydrogen. The technology has great advantages such as compact design, high current density (above 2 A cm^−2^), fast response, small footprint, low operating temperatures (20–80 °C) and the ability to produce ultrapure hydrogen [23]. However, high energy consumption and low hydrogen evolution rate are two important issues that hinder the large diffusion of this technology. Therefore, in order to increase the efficiency and reduce the energy consumption and costs, many researchers have dedicated their work to the development of alternative low cost materials, and efficiency increase and energy reduction [24]. Although the main challenging issue of the PEMWE technology is the enhancement of oxygen evolution rate, the improvement of membranes with reduced cost, appropriate conductivity, low gas cross-over and enhanced mechanical properties at high operating pressure is mandatory [25].

#### 1.1.4. A Short Review on PFSAs (Nafion Polymers)

**Figure 1:**
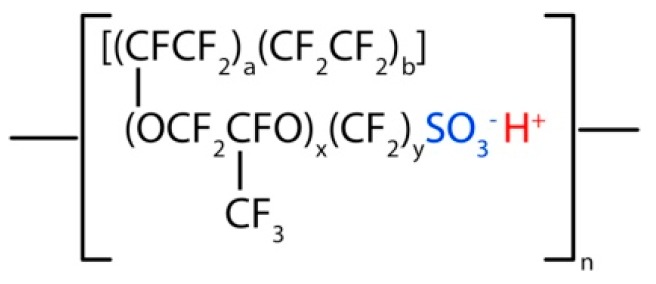
Nafion polymer structure; reproduced with permission from [26].

Nafion is a fluoropolymer made by sulfonated polytetrafluorethylene introduced by DuPont in the mid-1960s. It is commercially available with a thickness between 25 and 250 µm. As for Teflon, its structure (Figure 1) consists of CF_2_ (difluorocarbene) radicals, alternated to CFOCF_2_ and with end chains of sulfonic acid SO_2_OH. 

Main Nafion characteristics are the following:

- High proton conductivity: when the pendant sulfonyl fluoride groups (SO_2_F) are chemically converted to sulfonic acid (SO_3_H), the ionic functionality is introduced [27];
- Water permeability;
- High chemical resistance: only alkaline metals as sodium can damage the Nafion at STP (standard temperature and pressure); and
- High electronic resistance: forcing electrons to flow through the external circuit to generate electrical power.

The most important parameter to control, in order to keep the ion conductivity high is the relative humidity. Nafion membrane requires water to facilitate the transport of protons through either the Grotthus (hopping) mechanism or the vehicle (diffusion) mechanism [28]. This requirement has led to limiting PEMFC operating temperature to 80 °C as operation above that will lead to dehydration of the membrane and a subsequent loss in proton conductivity and fuel cell performance. However, PEMFC operating above 80 °C can benefit from increased reaction kinetics, reduced CO poisoning and simplified water and thermal management [29]. One method utilised to allow that transition into intermediate temperatures is the use of filler materials within the polymer [30]. Alternatively, there are polybenzimidazole membranes that are doped with phosphoric acids that operate at even higher temperatures [31].

Regarding the use in DMFC, Nafion has high ionic conductivity and chemical stability but high methanol permeability due to:

1. active transport of protons and water;
2. diffusion through the water-filled pores within the Nafion-structure;
3. diffusion through the Nafion itself.

Methanol crossover is an issue that leads to the much worse DMFC performance when compared with H_2_ PEMFCs. Various approaches to minimize or eliminate methanol crossover have been carried out: increasing membrane thickness [32], increasing the cathode reactant pressure [33], decreasing both cell temperature [34] and methanol concentration [35]. Another innovative way is to modify the membrane using materials that allow obtaining the same chemical and thermal characteristics of Nafion but with lower crossover and cost.

Also, in PEMWE, the commercial Nafion membrane is commonly used as a solid electrolyte due to its chemical and thermal stability, good proton conductivity, and mechanical strength [36]. A drawback of membranes made of Nafion is that they are known to lose water, and thus ionic conductivity, at temperatures above 100 °C [37], which prohibits them from being used for higher temperature water electrolysis. However, high operating temperature offers several advantages from enhanced electrode kinetics and reduced overpotentials [38]. Moreover, higher operation pressure would also be favourable for PEM electrolysis since it would reduce the gas pressurization constraints for storage purposes [39]. Hence, it would be important to develop membranes that can sustain high performance at higher operating temperature and pressure [40].

To overcome the drawbacks of Nafion membranes, novel membranes have been developed and can be classified into three main categories, namely; (i) polymeric, (ii) ceramic, and (iii) composite membranes.

Among these three categories, composite membranes have generated great attention recently. A composite (or hybrid) material can be defined as a material that includes two or more blended compounds on the molecular scale [41]. The use of filler material mixed into the Nafion (or an alternative ionomer) can aid in providing additional properties such as mechanical reinforcement, chemical resistance and proton conductivity. For example, hydrophilic fillers would result in increased membrane water uptake, ideal for low relative humidity (RH) operation. These filler materials can also be functionalised to provide secondary functionalities (e.g., sulphonating a hydrophilic filler) or boost the functionality it already has. Another example is the use of cerium oxide as a radical scavenger to slow down membrane degradation [42]. This review paper aims to provide a summary and analysis of the published work focusing on the development of composite membranes to improve the performance of the DMFC, modify operating conditions and enhance durability for PEMFC and PEMWE. 

## 2. Composite Membranes for DMFC

In this part, the range of composite membranes that have been developed to improve the performance of the DMFC at low temperature with reduced methanol crossover and low cost is reviewed.

Two categories of composite membrane materials are considered in the literature; modified Nafion; and non-perfluorinated polymers.

### 2.1. Composite Nafion-Based Membrane

Composite Nafion membranes can be loaded with organic and inorganic fillers that have been used predominantly to increase proton conductivity and to act as a barrier to methanol crossover [43,44]. The following sub-sections discuss the latest developments in the organic, inorganic and carbon nano-material filler-based membrane.

#### 2.1.1. Organic Fillers

Organic materials are commonly used as fillers in the polymeric composite membrane for fuel cells. They supply reinforcement and allow higher stability of the polymer matrix while making it more cost-effective. One of the most commonly applied organic filler is polytetrafluoroethylene (PTFE). PTFE is highly hydrophobic and although it is not suitable alone for membrane application for fuel cells [45], it can be used as a reinforcement of Nafion membrane due to its chemical stability, corrosion resistance and mechanical strength [46]. Few papers focused on testing DMFC performance using Nafion/PTFE membranes. Lin et al. [47] conducted a study on the application of this composite membrane, the authors investigated the effect of this polymer on conductivity, methanol crossover, and cell performance, and compared them with that of commercial Nafion membrane. Experimental data indicated that introducing PTFE into the Nafion polymer reduced both methanol diffusion and methanol electro-osmosis crossover in the membrane. The comparison between Nafion 117 and Nafion/PTFE was performed in a DMFC at 70 °C: Nafion/PTFE membrane was able to operate in a wider current density range achieving a maximum power output of 87.5 mW cm^−2^, 1.3 times higher than Nafion 117. This positive effect of the Nafion/PTFE membrane was also obtained by Nouel et al. [48] and Yu et al. [49] who tested a fuel cell MEA made of Nafion/PTFE comparing results with Nafion 117, 115 and 112. The performance was higher than Nafion 117 and 115 but similar to 112. 

In an attempt to further enhance the performance of Nafion/PTFE membrane, Chen et al. [50] included zirconium phosphate (ZrP) into the membrane structure and so the Nafion matrix was modified with both PTFE and ZrP/PTFE for comparison. The composite membranes were prepared via two processes:

1. By impregnating PTFE directly in a Nafion/ZROCL_2_ solution and then annealing it at high temperature;
2. By impregnating the PTFE membrane in a Nafion solution, annealing at high temperature to prepare Nafion/PTFE membrane, then impregnating again in a ZrOCL_2_ solution.

Experimental results indicated that the introduction of ZrP led to reduced methanol crossover and proton conductivity. The impact of proton conductivity is stronger than methanol crossover on DMFC performance, thus, as confirmed by tests conducted on the cell, the performance of ZrP/PTFE was lower than Nafion/PTFE.

Most research is focused on the preparation and modification of various proton conductive membranes that are inexpensive and provide better performance and properties than Nafion membranes. To this end, innovative organic materials, which have good thermal and chemical stability and can be easily modified to be used as ionic conductive membranes such as polybenzimidazole (PBI) and polyvinyl alcohols (PVA), were studied [51,52].

Shao et al. [53] and Mollà et al. [54] fabricated Nafion/PVA membranes using casting [55] and impregnation method [56,57] respectively. PVA has higher affinity for water than to methanol (i.e., 55 wt. % and 10 wt. %, respectively), so it can be potentially used for DMFC applications. Both works demonstrated that comparable DMFC performance can be obtained using these membranes. Specifically, Mollà et al. focused on the characterization of Nafion/PVA membranes with varying operating temperature (45, 70 °C), thickness of the membrane (19–47 µm) and concentration of methanol (1–2M). The performance of pristine Nafion membrane and Nafion/PVA were roughly equivalent at very low thickness while Nafion/PVA exceeded the pristine Nafion performance only at higher thickness and higher temperature. At any fixed condition; thickness, temperature and methanol concentration, the OCV of Nafion/PVA was higher than pristine Nafion indicating reduced methanol crossover.

Hobson et al. [58] presented Nafion-PBI dipped and screen-printed films to investigate the effect on membrane performance. They concluded that the modification of Nafion with PBI by both spin coating and dipping reduced the methanol permeability; however, the benefit of low methanol crossover was counterbalanced by the negative effect of the too high impedance. Since neither of the techniques produced a suitable membrane for DMFC, screen printing was investigated and here methanol permeability was reduced without an increase in impedance. The membranes were then tested in a single cell at 60 °C. Using methanol solution of 3.2 M, the cell performance was greatly improved with the current density increased by 42% combined with an increase in maximum power output by 46% as compared with the pristine Nafion membrane. Ainla et al. [59] work on Nafion-PBI membrane was in agreement with the above results. In fact, they demonstrated that the Nafion-PBI membrane has lower methanol permeability and higher conductivity than a commercial membrane. It is important to note that the utilization of these composite membranes led to lower methanol permeability and enhanced the performance only at high methanol concentration.

Conductive Polymers such as polyaniline (PANI) and polypyrrole (PPy) have recently been incorporated into Nafion membranes to reduce its methanol permeability [60,61]. Composite Nafion polypyrrole membranes were prepared by two methods: electrodeposition of polypyrrole on Nafion-coated electrodes [62] or by in situ polymerization with a chemical oxidant [63]. Zhu et al. [64] made a membrane by in situ polymerization using Fe(III) and H_2_O_2_ as oxidising agents. The electrostatic interaction between the sulphonate groups of Nafion and polypyrrole, decreased the pore volume of Nafion membrane which led to low methanol permeability. However, the electrostatic interaction between the polypyrrole chains and sulphonate groups of Nafion also decreased the proton conductivity and therefore increased membrane and cell resistances. So, the benefit of the reduced methanol crossover was neutralized particularly when DMFC worked at high current densities. 

Polyaniline is a good conductive polymer that can improve both methanol oxidation and the stability of the catalyst; it can also be included in the Nafion structure through both electrochemical and chemical modification to improve its properties. Wang et al. [65] and Escudero-Cid et al. [66] assembled a composite membrane of Nafion/polyanilina and carried out DMFC performance tests including polarization curve and durability tests showing that both the ionic conductivity and methanol permeability of the Nafion membrane containing PANI decreased when compared with Nafion membrane. In particular, Wang et al. indicated that the performance of the fuel cell increased using the modified membrane especially at high methanol concentration (maximum power output at 6 M) while the power output using Nafion pristine membrane was reduced with increasing methanol concentration. Moreover, it was noted that the PANI composite membrane performed better than that with polypyrrole [67]. It is important to highlight here that the reviewed papers; investigating the use of conductive polymers, do not consider the change in the electronic conductivity of the membrane (short circuit current) in the composite membrane. This should be considered in any future work on these materials.

In recent years, two types of sulphonate fillers, sulphonate poly arylene ether ketone (SPAEK), and sulphonate poly ether ether ketone (SPEEK) have been developed and used to modify the Nafion membrane in DMFCs. Both SPAEK and SPEEK have good attributes: high proton conductivity and methanol resistance for SPAEK [68,69]; good mechanical properties, proton conductivity and good processing capacity of SPEEK polymers [70,71]. Regarding the behaviour in a methanol fuel cell [72,73], an increase by at least 30% in OCV and by 10% in highest power density were observed. The positive results suggest that these membranes could be taken into account for use in future DMFC application once durability is deeply investigated.

#### 2.1.2. Inorganic Fillers

The implementation of inorganic particles into Nafion helps enhance the thermal stability and proton conductivity of composite membranes making them more attractive and appropriate than bare Nafion membranes [74]. This section focuses on silica, metal oxides, montmorillonite and zeolite fluorinated composite membranes; materials that have received considerable attention due to their chemical and thermal properties.

Silica materials have attracted a considerable attention because they possess high surface area and high chemical stability [75]. Generally, they are prepared using different precursors such as alkoxy silanes (like tetraethyl orthosilicate (TEOS)), sodium metasilicate and fumed silica [76]. The addition of silica compounds into polymer membrane is believed to lower methanol crossover [77,78]. In this context, Ren et al. [79] prepared a composite membrane modifying Nafion polymer with tetraethyl orthosilicate (TEOS) and sulfonic TEOS using the casting method. They investigated the influence of silica into Nafion matrix and the changes in proton conductivity, methanol permeability and performance. The results showed that the proton conductivity of these composite membranes was lower than that of commercial Nafion membranes due to the hydrophobic side chain of the TEOS that reduced the water content of the membrane. However, the methanol permeability was also reduced. DMFC single cell tests were carried out at both 1 M and 5 M and at 75 °C. The polarisation curves depicted how the silica composite membrane could achieve better performance than the Nafion when using high methanol concentration because although the proton conductivity of the composite membrane decreased, the methanol permeability also reduced. Works are necessary to increase proton conductivity of those composite membranes.

Some studies have been carried out to investigate experimentally the effect of Nafion membranes with metal oxides, such as; SiO_2_, TiO_2_, WO_3,_ as fillers on the performance of DMFCs [80]. As a result of these experiments, the Nafion-modified composite membranes provided higher power density in comparison to the commercial Nafion 115 membrane. Regarding the application in a DMFC, Nafion/TiO_2_ improved water uptake and reduced methanol absorbance [81] while Nafion/SiO2 showed lower methanol permeability than commercial membrane [82]. Moreover, Nafion membrane modified with both SiO_2_ and TiO_2_ were prepared by solvent casting method and studied by Ercelik et al. [83] that investigated the effect of these particles on proton conductivity, water uptake and performance varying temperature. The authors claimed that:

- proton conductivity of Nafion-TiO_2_/SiO_2_ increased with temperature. The maximum value obtained was 0.255 S cm^−^^1^ which is 25% higher than Nafion 117 at 75 °C;
- water uptake values are similar with those of bare Nafion; and
- power densities of MEAs with composite membranes are higher than the MEAs using Nafion 115: at 80 °C and 1M of methanol concentration. The maximum power densities obtained by Nafion/TiO_2_, Nafion/SiO_2_ and by the MEA with bare Nafion are 641.16 W/m^2^, 628.68 W/m^2^ and 612.96 W/m^2^, respectively.

The abovementioned studies demonstrated that the incorporation of inorganic particles like SiO_2_ and TiO_2_ provided better performance if compared with the Nafion membrane. Although, the use of metal oxides as filler has enabled many advantages, they too have some problems associated with them. The metal particles are often very difficult to disperse homogenously in the polymer membrane, it would mean that the performance of the composite will not be uniform throughout the bulk of the membrane. Moreover, metal oxides accelerate the degradation of membranes, and so durability studies are required to understand the actual benefits and drawbacks of these fillers.

Montmorillite (MMT) is a filler that has attracted much attention recently as Nafion/MMT membranes have been reported to have improved mechanical and thermal properties compared to pristine Nafion membranes [84]. But, the incorporation of this filler into the Nafion matrix does not improve the proton conductivity. Wu et al. [85] prepared the composite membrane via casting solution and reported a slight decrease (about 9%) in proton conductivity compared with pristine Nafion but the methanol crossover decreased more than 90% by loading MMT of only 1% wt [86]. As described above, the utilization of this filler does not contribute to improve the proton conductivity, thus, to minimize the loss in performance Rhee et al. [87] and Lin et al. [88] modified the montmorillonite with an organic sulfonic acid group (MMT-SO_3_H) with varying the content of the filler. Their studies showed that the proton conductivity of the composite membranes generally declined from that of pristine Nafion membrane with the increase in the inorganic filler content, but the methanol permeability was reduced by up to 90%. The combination of these effects led to an improvement in the performance of a DMFC. In fact, the polarization curve of the MEA with Nafion 115 and composite membrane, realized at 40 °C and 2M of methanol concentration, showed that the performance of the DMFC improves initially with increasing the inorganic content, with a maximum power density at 5 wt. % loading. Curves revealed that all composite membranes achieved better performance than Nafion membrane at high current density region. However, the thermal stability of the membrane is not yet adequate and performance at higher temperature and methanol concentration deserves to be investigated.

To hinder permeation of methanol, another approach is to develop composite membranes using zeolites. Zeolites are micro porous crystalline materials containing silicon, aluminium and oxygen in their framework. They are based on an infinitely extending three-dimensional network of AlO_4_ and SiO_4_ tetrahedra linked by sharing oxygen atoms [89,90]. The chemical structural formula of a zeolite may be expressed by the following [91]:

Mx/n[(AlO_2_)_x_(SiO_2_)_y_]m H_2_O (10)

where M is a cation of valence n, m is the number of water molecules and the sum of x and y is the total number of tetrahedra in the unit cell.

Several authors [92,93] have claimed that zeolite membranes can be adopted for DMFC application. The approach of these studies was to take advantage of the molecular sieving property of zeolite to prevent methanol from passing through the membrane. However, a pure zeolite exhibits poor mechanical properties such as brittleness and fragility and hence is unsuitable for use as a membrane. Moreover, the performance of the zeolite composite membranes depends on the zeolite properties in terms of pore size and surface tension (hydrophobility or hydropholicity). It was reported that hydrophobic zeolites ensure low affinity to water so high permeability to methanol however hydrophilic zeolites lead to an opposite trend and therefore reduce methanol crossover [94]. When zeolites are combined with a polymer support (e.g., Nafion), the advantages of both polymer and zeolite are combined. Among the various type of zeolites, mordenite (MOR) and analcime (ANA) have attracted a lot of interest because they are hydrophilic substances which promote the adsorption of water, excluding alcohol, and provide a good proton pathway through the membrane. Prapainainara et al. [95] fabricated composite membranes with those two fillers studying and comparing their properties and performance. The authors claimed that the presence of the filler benefited the proton migration through the membrane whilst the homogeneous distribution of the filler contributed to block the flow of methanol through the membrane, leading to lower methanol permeability. The composite membrane with MOR filler gave better membrane properties, namely; higher proton conductivity and lower methanol permeability, than those using ANA filler. The best DMFC performance was achieved by MOR composite membrane with a maximum power density of 10.75 mW/cm^2^, 1.5 times higher than ANA membrane and two times higher than a commercial Nafion 117 membrane. 

To enhance the performance of MOR/Nafion, Prapainainara et al. [96] incorporated graphene oxide (GO) to the matrix. The authors used GO to modify the surface of MOR by increasing the surface hydrophilic functional groups resulting in better incorporation of MOR to Nafion and comparable chemical properties with those of pristine Nafion and MOR/Nafion. The use of GO led to better proton conductivity, 1.5 times higher than that of Nafion/MOR and Nafion 117 at 70 °C and it had the lowest methanol permeability too. The authors also tested the membrane in a single cell, obtaining a power density of 27.5 mW/cm^2^; almost 5 times than that of Nafion 117 at the same operating condition (1 M methanol, 70 °C). However, the operation lifetime was still not good enough for commercial applications.

#### 2.1.3. Carbon Nanomaterial Fillers

Graphene oxide (GO) was used as a filler in Nafion membranes because it allows easy proton transport and good water uptake due to its high surface area. The different oxygen groups such as epoxide, hydroxide, carbonyls and carboxyls converts GO into electrically insulating and hydrophilic material [97] while retaining other properties like mechanical strength, surface area, and gas impermeability.

**Figure 2:**
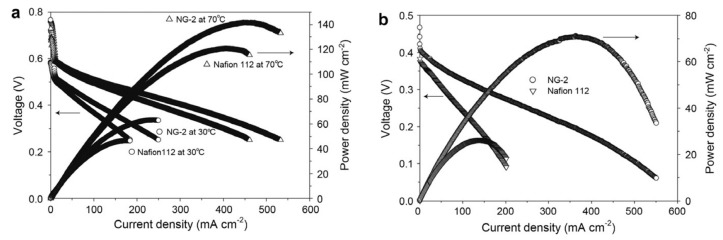
Polarization curves of DMFC obtained for Nafion 112 and GO composite membranes at (**a**) 1M methanol at 30 °C and 70 °C and (**b**) 5M methanol at 30 °C; reproduced with permission from [98].

Graphene oxide has an excellent compatibility with Nafion so it can be adopted as a modifier to improve the selectivity (to allow the passage of specific species) and performance of such membranes. Choi, et al. [98] developed a composite GO/ Nafion membrane. The authors claimed that the compatibility between both components was guaranteed due to their strong interfacial attraction. GO enhanced thermal backbone and side chains stabilities due to the interaction between GO sheets and Nafion: the non-polar backbone of Nafion interacted with the hydrophobic structure of GO while the polar ionic clusters of Nafion with the hydrophilic groups of GO. Their study revealed that the permeability for methanol with just 0.5 wt.% of GO was reduced to 60.2% of Nafion 112 at 25 °C. However, the proton conductivity tests revealed an opposite trend showing a decrease with increasing the GO filler content and a loss of 55.3% in proton conductivity is reported with 2 wt. % GO loading. This was not completely unexpected as GO alone is not known to be an excellent proton conductor. The authors obtained the maximum power density of 62 mW cm^−2^ at 30 °C and 141 mW cm^−2^ at 70 °C after optimising the GO loading in the membrane (1% wt.) as depicted in Figure 2.

Moreover, at high methanol concentration, where methanol crossover becomes critical, benefits provided from the incorporation of graphene oxide were more evident: the composite membrane showed much higher power density, 3 times higher than Nafion (71 mW cm^−2^ vs 26 mW cm^−2^).

Chien, et al. [99] prepared a composite membrane with sulphonated graphene oxide (SGO)/Nafion for DMFC to avoid the aggregation of GO in the polymer matrix. It was reported that proton conductivity increases with increasing amounts of SGO, as the SGO was distributed throughout the matrix and created more interconnected transfer channels. However, with further SGO amounts, aggregation began to predominate, thus reducing the conductivity of the composite membrane. Methanol permeability was shown to decrease in the presence of SGO as they block the methanol migration through the membrane. In DMFC test, the SGO/Nafion composite membrane exhibited higher current and power densities than commercial Nafion 115, for example;

- in 1 M methanol solution, the current density and power density for the composite membrane at 0.4 V were 102.7 mA cm^−2^ and 42.9 mW cm^−2^, whereas the commercial Nafion 115 revealed only 78.6 mA cm^−2^ and 32.6 mW cm^−2^.
- in 5 M methanol solution, the composite membrane showed values of 83.2 mA cm^−2^ (at 0.4 V) and 34.6 mW cm^−2^, which were better than the commercial membrane (54.1 mA cm^−2^ at 0.4 V and 22.1 mW cm^−2^).

Additionally, the SGO/Nafion composite membrane had a lower catalyst activation loss than Nafion 115, which indicated that the composite membrane had lower methanol crossover and faster reaction kinetics.

Yan et al. [100] proposed an innovative way to modify Nafion membrane by sandwiching a graphene oxide layer between two Nafion membranes. With the addition of a monolayer graphene film, methanol permeability decreased by 68.6% while observing only a marginal decrease in proton conductivity of 7% at 80 °C in comparison to pristine Nafion membrane. The authors tested the membrane in a DMFC varying methanol solution from 5 M to 15 M. Tests depicted that the graphene film allowed for a substantial performance improvement particularly when the passive DMFC was fed with high concentration methanol solutions enabling the passive DMFC to be operated at high concentrations.

### 2.2. Non-Perfluorinated Polymers Composite Membranes

Non-fluorinated membranes seem to have a promising future for DMFCs as a replacement for the expensive fluorinated membranes that have high methanol and ruthenium crossover. Aromatic polymers are considered to be one of the most promising routes to obtain high performance polymer electrolytes because of their availability, variety of chemical composition and stability in the cell environments. Specifically, poly ether ether ketone, polyvinyl alcohol, and poly arylene ether and their derivatives are currently under investigation.

Poly(ether ether ketone)s (PEEKs) [101] are semicrystalline polymers that present high thermal and chemical stability. The sulfonation of PEEK produces copolymers with sulfonic acids into the aromatic backbone; membranes made of these sulfonated polymers show useful properties for DMFCs, such as; low methanol cross-over, good ion conductivity, thermal stability and high mechanical strength [102]. The proton conductivity of SPEEK depends on the sulfonation degree [103], it generally increases with the sulfonation degree but high sulfonation results in high methanol permeability so that its application is limited [104]. The development of SPEEK composite membranes is currently being investigated by using SPEEK for the polymer matrix and modifying it in order to reduce methanol permeability at high sulfonation degree. Many researches have focused on SPEEK-based membrane with phenoxy resin [105], polyphenylsulfone (PPSU) [106], solid heteropolyacids [107], polyaniline [108], SiO2 and zirconium phosphate (ZrP) [109], zeolite [110], polypyrrole [111]. As discussed above, the utilization of montmorillonite and polybenzimidazole into Nafion matrix improved the DMFC performance; in the same way, they can be used to modify the matrix of SPEEK polymer. In fact, Gosalawit et al. [112] used a SPEEK/MMT membrane in their work and compared its performance in a DMFC single cell with pristine SPEEK membrane and Nafion bare membrane. The study confirmed that the performance was higher: current density generated from the MEAs of Nafion 117, SPEEK, SPEEK/MMT 1wt.%, SPEEK/MMT 3wt.% and SPEEK/MMT 5 wt.% membranes at the constant voltage of 0.2 V were 51, 76, 103, 96 and 94 mAcm^−2^, respectively with the maximum power density of 10, 15, 21, 19 and 18 mWcm^−2^. However, the thermal stability was significantly reduced. Pasupathi et al. [113] synthetized a non-perfluorinated membrane by casting SPEEK and PBI solution into a glass plate. A SPEEK/PBI membrane enhanced DMFC performance: the maximum power density obtained (45 mW cm^2^) was two times higher than Nafion 117 at 60 °C. Moreover, SPEEK/PBI membranes were found to be extremely stable under DMFC operating conditions up to 60 °C. However, their stability dropped considerably at higher temperatures. Experiments are underway to address the stability issue of these membranes at higher temperatures.

Generally, sulfonated aromatic polymer membranes require a high sulfonation level to achieve sufficient proton conductivity resulting from the low acidity of the sulfonic groups in the aromatic rings [114]. However, such a high sulfonation level usually makes them excessively swell and even soluble in methanol/water solution which may lead to a loss in mechanical properties and become unavailable in applications [115]. Therefore, they should be modified including organic or inorganic fillers. Jiang et al. [116] investigated the performance of a SPEEK/GO membrane in which GO is sulfonated (SGO) to improve the proton conductivity of the membrane. In fact, the SPEEK/GO membranes exhibit lower ion exchange capacity (IEC) and water uptake than Nafion membrane, and the overall proton conductivity of the membranes remains low. This is due to the lack of proton conductive groups on the pristine GO. Therefore, these membranes are still not quite suitable for use as PEMs in DMFCs. However, by using higher contents of sulfonated GO, these SPEEK/SGO membranes showed even higher IEC and proton conductivity compared to Nafion 112, which makes them particularly attractive as PEMs for the DMFC applications. It is worth noting however that the swelling ratio of membranes increased with the increase of the content of the SGO. DMFCs with SPEEK/SGO showed better performance than those with the plain SPEEK or the pristine SPEEK/GO. With the optimized contents of SGO in SPEEK (3% and 5% wt.), the DMFCs exhibited 38 and 17%, respectively, higher performance than those with Nafion 112 and Nafion 115. Despite of having higher IECs and proton conductivity, the membranes with higher content of the SGO exhibited higher methanol permeability leading to a decrease of the fuel utility and the lifetime of the cathode catalysts, thus low performance. In this regard, for practical applications of the SPEEK/SGO membranes, the contents of SGO in the SPEEK matrices should be well controlled.

Sulfonated Poly (arylene ether sulfone) membranes (SPAES can be useful for methanol fuel cell through the modification of the polymer matrix by introducing inorganic/organic particles such as laponite. Laponite is made of silica tetrahedral and alumina octahedral sheets which have advantageous hygroscopic properties [117]. This inorganic compound was used by Kim et al. [118] to prepare and evaluate the behaviour of the SPAES membrane. Properties of the hybrid membranes for DMFC such as methanol permeability, and proton conductivity were investigated. Authors claimed that methanol permeability was lower than that of a SPAES pure membrane and Nafion using membranes with a small content of Laponite. This is because the presence of Laponite improved the barrier property of the membrane to methanol molecules. This was likely due to the tortuosity of layered silicate and the lower aspect ratio of the particles resulting from their exfoliation increasing methanol diffusion paths through the composite membrane. However, the proton conductivity was very low and further research should be conducted to optimise Laponite loading.

Another approach to reduce the undesired swelling property and methanol crossover of sulfonated membrane is to crosslink membranes. This method has been widely investigated by many researchers for crosslinking SPEEK membranes [119,120]. These membranes showed decreased swelling ratio and methanol crossover but decreasing proton conductivity. Following this method, Feng et al. [121] used sufonated poly (arylene ether)s as PEM materials due to their good thermal stability, high glass transistion and excellent mechanical strength. They synthesized sulfonated poly (arylene ether sulfone) copolymers with propenyl groups then crosslinked using benzoyl peroxide varying the crosslinking. Proton conductivity and methanol permeability were evaluated and compared with Nafion 117, and showed that:

- the proton conductivity of the SPAES membranes increased from 0.1 to 0.16 S/cm with the increase of temperature from 30 to 70 °C which was quite similar to that of Nafion 117: 0.11 to 0.17 S/cm from 30 to 70 °C [122];
- the methanol permeability evaluated was lower than that of Nafion117 (2.07 × 10^−6^ cm^2^/s): the less pronounced hydrophobic/hydrophilic separation of sulfonated polyaryls compared to Nafion corresponded to narrower, less connected hydrophilic channels and larger separations of the less acidic sulfonic acid functional groups, which affected the permeability of methanol [123].

Poly vinyl alcohol (PVA) is usually synthesized from poly vinyl acetate and commonly used for adhesive, food wrapping, and desalination and pervaporation membranes [124]. Regarding the possible use of a membrane made of PVA in DMFC, it is known that PVA does not have any negative charged ions, like carboxylic and sulfonic acid groups, so the conductivity is very low as compared with Nafion membrane. Therefore, PVA membranes can be used in a fuel cell if negative ions are incorporated within their structure to increase their conductivity [125]. Moreover, several studies have shown how PVA polymer membrane leads to a reduced methanol crossover [126]. Regarding the reduction of methanol crossover, it was demonstrated that the addition of filler into PVA matrix contributes to mitigate this issue: fillers such as SiO_2_ [127], polyrataxane [128] were reported. Yang et al. [129] used montmorillonite (MMT) as a filler and tested ion conductivity, methanol permeability, current density-potential and power density curves of the PVA/MMT composite polymer showing the following properties:

- high ionic conductivity: 0.0368 S cm^−^^1^, performed by PVA/10wt. % MMT at 30 °C;
- methanol permeability: 3–4 × 10^−^^6^ cm^2^ s^−^^1^, which was lower than that of Nafion 117 membrane of 5.8 × 10^−^^6^ cm^2^ s^−^^1^;
- maximum peak power density: 6.77 mW cm^−2^ at ambient pressure and temperature with the PtRu anode based on Ti-mesh in a 2M H_2_SO_4_ + 2M CH_3_OH solution.

### 2.3. Other Composite Non-Fluorinated Membranes

The modified polyvinylidene fluoride (PVDF) membranes using inorganic additives were prepared with a view of combining the properties of inorganic ion exchanger (high thermal stability, and excellent water holding capacity at higher temperatures) and organic support (chemically stability and high mechanical strength). Impregnation of porous polymeric film of PVDF is the method used by Pandey et al. [130,131] to synthesize PVDF/silica and PVDF/Zirconium phosphate (ZrP). Single cell DMFC tests were carried out to study the DMFC performance for the synthesized membrane. The membranes showed better thermal stability, water uptake ratio and lower methanol crossover than Nafion 117, however, performance were low because of poor proton conductivity.

PolyFuel Inc. produced polycarbon membrane for passive DMFC [132] showing a power density of 80 mW cm^−2^ for thickness of 45µm, lifetime for a nearly constant runtime is 5000 h and back diffusion of water was improved by 30%, which helped mediate the dissolution of the methanol concentration in a passive DMFC.

There are also other composite membranes developed for PEMFC applications which may also have a good prospect for DMFC. These include trifluorostyrene-based membranes developed by Ballard Power System Inc [133], a butadiene/stryene rubber-based membrane developed by Hoku Scientific Inc [134] and polystyrene sufonate (PSS) membranes [135]. Table 1 summarizes the properties and the pros and cons of composite electrolyte membranes described in this review compared to those of the commercial membrane. In addition, Table 2 summarizes their DMFC best performance.

According to the data collected into the two tables above, membranes with fillers guarantee the highest performance. This is due to the fact that the reduced alcohol permeability counterbalances the reduced proton conductivity in the composite membranes. Materials, such as; PTFE, PVA and metal oxided are not proton-conducting materials so result in a reduction in the electrolyte proton conductivity, however they increase the tortuosity of the membrane thereby leading a reduced amount of crossover. Two approaches to increase the proton conductivity were adopted to further enhance the performance, namely; by optimising the filler content or by functionalising the filler (most commonly by incorporating sulphonic groups) to increase the overall electrolyte proton conductivity.

Polarization curves, proton conductivity, water uptake and methanol crossover are tests commonly carried out for all the membranes described in this section. However, durability tests are still lacking in the literature. Therefore, only commercial Nafion provides guarantees in terms of lifetime and degradation, therefore it cannot be completely substituted up to this time. Research activities on the lifetime time and the degradation of Nafion based composite membranes should be carried out.

## 3. Composite Membranes for H_2_ PEMFCs

### 3.1. Inorganic Fillers

**Figure 3:**
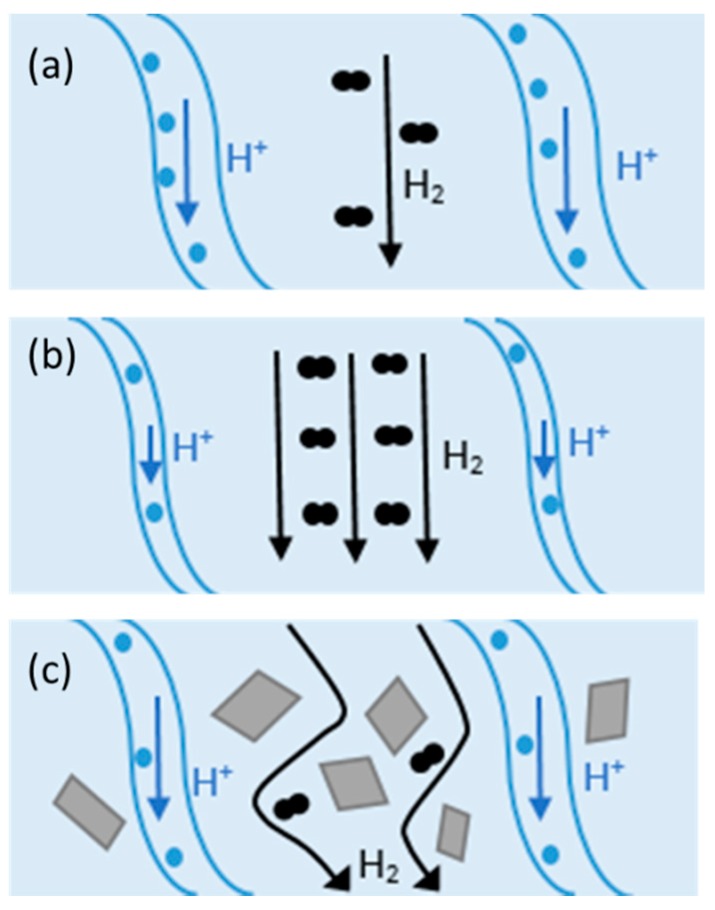
(**a**) Hydrogen and proton transport through Nafion at 80 °C (**b**) Hydrogen and proton transport through Nafion at higher temperatures (**c**) Hydrogen and proton transport through Nafion composite at higher temperatures.

Inorganic fillers have a long history of use as fillers in membranes for fuel cells. The general explanation of their suitability is due to their high thermal stability, mechanical strength, and water-absorbing nature. Therefore, the main aim of introducing fillers into the polymer membrane is to enhance its properties and enable its operation at elevated temperatures and/or low relative humidities. Figure 3 illustrates the change in proton conductivity and hydrogen crossover with the change in operating conditions to higher temperatures and lower relative humidities (Figure 3b) and the incorporation of fillers (Figure 3c).

At 80 °C and with high relative humidity, the membrane and its channels are fully saturated with water. The protons travel through the membrane via either Grotthus mechanism or via diffusion. There is also molecular hydrogen that passes through the membrane, known as hydrogen crossover, that then interacts with the cathode resulting in a reduced OCV.

At higher temperatures, the water within the membrane begins to evaporate. This results in a shrinkage in the channels within the membrane and less water for protons to either hop across or diffuse through the membrane, resulting in a subsequent drop in proton conductivity. Furthermore, the effect of hydrogen crossover is enhanced due to the increased operating temperature. 

The addition of a filler material increases the path of the hydrogen to pass from the anode to the cathode. This increased tortuosity results in a decrease in hydrogen crossover. Also, the filler material itself can be functionalised, so for example, hydrophilic fillers can draw lots of water, hence improving proton conductivity and reducing the detrimental effects of increased temperatures.

Di Noto et al. studied the proton-conducting properties of mixed organic-inorganic membranes with Nafion mixed with various metal oxides, such as titanium, zirconium, hafnium, tantalum and tungsten oxides [136]. Thermal experimentation revealed that the composite membranes are stable below 170 °C, suggesting their possible application in PEMFCs that operate at elevated temperatures. Four different water domains were detected in the composite membrane, regarding the different interactions such as bulk water and water solvating ions interacting with the sulphonic side group. The quantity of each domain depends on the filler material. The authors proposed that the conduction mechanism within the studied membranes involves proton hopping through different fluctuating water domains [137].

Adjemian et al. [138] also investigated composite membranes with different metal oxide fillers such as titanium, silica, alumina, and zirconium for PEMFCs with varying the operating temperature from 80 to 130 °C. It was found that the membranes with titanium oxide or silica revealed better performance at higher temperature and lower humidity conditions compared to recast Nafion. Furthermore, a study into carbon monoxide tolerance revealed that; by using the composite membranes and increasing the operating temperature to 130 °C, the CO tolerance of the catalyst layer was improved to 500 ppm of carbon monoxide without failing comparted to 50 ppm at conventional operating conditions. In this study, none of the composite membrane filler materials were surface modified so potentially further performance enhancement can be achieved by functionalising the fillers.

A composite membrane with Nafion and a filler consisting of silica nanoparticles with surface modified fluoroalkyl functionalities was presented in [139]. It was noted that although the silica nanoparticles are hydrophobic, the water uptake of the membrane was not negatively affected. In addition, the composite membrane showed thermal stability up to temperatures of 240 °C. Proton conductivity tests revealed that the composite membrane with 5 wt. % silica nanoparticles with a ratio of [Nafion/ (Si80F) 0.7] had the highest conductivity at 0.083 S cm^−^^1^ at 135 °C. Following from this, a single cell test was performed with the composite membrane at 85 °C. The composite membrane displayed a better power density compared to the recast Nafion, when the oxidant is air and oxygen (Under air: 0.38 vs 0.27, under oxygen: 0.48 vs 0.35 W cm^−2^ composite and recast Nafion respectively). It would be interesting to see the behaviour of the membrane at elevated temperatures. 

Following on from their work, Griffin et al. [140] fabricated and characterised a composite membrane with sulphonated zirconia dispersed in a Nafion matrix. The idea behind this is that functionalising the zirconia with sulphonic groups would boost the proton conductivity of the membrane. Proton conductivity tests at 120 °C and under anhydrous conditions revealed that the membrane had a conductivity of 3 × 10^−^^3^ S cm^−^^1^. This makes the membrane ideal for fuel cell applications at intermediate temperatures and under dry condition. 

Saccà et al. [141] studied the influence of zirconium oxide as a filler material at different loadings of 5, 10 and 20% for Nafion composites. Recast Nafion membranes had a water uptake of 20%. The addition of zirconium oxide led to an increase in water uptake to 24, 24, and 30% for loadings of 5, 10 and 20% respectively. Fuel cell testing of the membranes in a single cell at operating temperatures of 80, and 110 °C show that at 80 °C, addition of 5% of filler makes very little difference in performance compared to recast Nafion. However, the membrane with 20% filler had a much lower potential, potentially due to excessive water uptake at 80 °C. Composite MEAs with 10% filler produced higher polarisation compared to Nafion at both temperatures with a maximum power density of 400 mW cm^−2^ was achieved at 130 °C, 85%, and 0.5–0.6 V.

D’Epifanio et al. [142] took this one step further and sulphonated the zirconium oxide. Water uptake experiments at 25 °C with varying relative humidity revealed that both composite membranes outperformed recast Nafion at all relative humidities (30 to 100%). Polarisation curves at 70 °C and at three different RH (65, 83 and 100%), show that the composite membrane produced better current densities at all voltage ranges, with current densities of 1015 mA cm^−2^ vs 680 mA cm^−2^ at 0.6 V, respectively. The difference between the two polarisation curves was emphasized during the ohmic and mass transport region, showing that the filler reduced ohmic resistance and improved water diffusion. A final test at 30% RH showed even greater differences with current densities of 930 mA cm^−2^ vs only 200 mA cm^−2^.

Alberti et al. [143] attempted to improve the proton conductivity and stability of membranes at elevated temperatures and studied the effect of doping Nafion with zirconium phosphate. However, they found that the conductivity decreases with increasing filler loadings. In addition, the authors explained that the difference in proton conductivity between Nafion and their composite membrane is mostly at lower relative humidities and higher filler loadings. On the other hand, Sahu et al. embedded silica nanoparticles into Nafion via a sol-gel method [144]. Single cell tests at 100 °C and at 100% RH showed the composite membrane (doped 10 wt. % silica) produced a peak power density of 350 mW cm^−2^. Moreover, composite membranes with 15 wt. % experience large mass transport losses due to flooding. 

Costamagna et al. [145] then prepared zirconium phosphate Nafion composite membranes via the impregnation of Nafion 115 and recast Nafion for high temperature PEMFC use. The composite membrane from Nafion 115 produced a current density of 1000 mA cm^−2^ at 0.45 V and at an operating temperature of 130 °C, which is much better compared to 250 mA cm^−2^ pristine Nafion. In addition, the cell fabricated from recast Nafion reached current densities of 1500 mA cm^−2^ at the same operating conditions. 

Furthermore, Sahu et al. [146] presented a Nafion composite membrane with mesoporous zirconium phosphate as the filler, prepared via a co-assembly method. The single cell testing was performed at 70 °C and at varying relative humidities, 100, 50, 31 and 18%. The difference between the composite membrane and pristine Nafion membrane increases with decreasing RH (via the maximum power density peaks). In terms of filler loading, the best performing was 5 wt. %, followed by 10 and 2.5. At 18% RH, the composite membrane produced a maximum power density of 353 mW cm^−2^, in comparison to pristine Nafion’s 224 mW cm^−2^ (both at 500 mA cm^−2^).

Pineda-Delgado et al. [147] decided to study the behaviour and performance of Hafnium oxide Nafion composite membranes. The fabricated composite membranes displayed greater water uptake of 61% at 100 °C compared to 29% for recast Nafion. This improvement in water uptake led to better proton conductivity at 100 °C with 112 vs 82 mS cm^−^^1^, for the composite and recast Nafion respectively. Following from this, the authors decided to test their membranes in a single cell set up at operating temperatures of 30, 50, 80 and 100 °C. The recast Nafion achieved a greater maximum power density at 30 and 50 °C, but at 80 and 100 °C the composite membrane performed better. At 100 °C, the composite produced a maximum power density of 0.336 W cm^−2^ compared to 0.188 W cm^−2^ for the recast Nafion, at a voltage of 0.46 V.

The performance of sulphonated silica Nafion composites where assessed where the filler was synthesised with a simple sol-gel calcination process [148]. Optimisation studies revealed that a 1% filler was the optimum loading, outperforming 0.5, 1.5% and recast Nafion. In-situ fuel cell testing under reduced humidity also confirmed the initial ex-situ results. The authors attributed the enhanced performance to efficient proton transport due to the well-defined phases in the membrane structure which was seen with TEM. 

One method to improve the dispersion of filler material within the polymer matrix is to swell the polymer membrane in a solution of the filler [149,150]. Xu et al. employed this technique by swelling the Nafion membrane with silica to achieve a composite membrane, in comparison to the traditional solution casting technique. They highlighted that this method maintains the ordered nanophase-separation structure of Nafion. This was shown in water uptake tests, where the swelled composite showed a higher water uptake but lower swelling, in comparison to the recast membrane. Fuel cell testing at 110 °C and 20% RH showed that the swelled composite produced a maximum power density of 113 mA cm^−2^, in comparison to 80 mW cm^−2^ for recast Nafion with no filler. The performance was explained due to the lower internal resistance of the composite membrane.Saccà et al. [151] introduced titanium oxide of different loadings (5, 10 and 15 wt. %) into Nafion for the purpose of operating fuel cells at a reduced humidity. SEM images revealed that the dispersion of filler throughout the cross-section of the membrane show that the lower filler loadings are better dispersed. The higher loading membranes showed the presence of filler agglomerates. Water uptake testing at different temperatures showed that there is a small drop initially when the filler material is introduced. In addition, the higher loading membranes are less influenced by the increasing temperatures. A similar trend was also observed for swelling, with the composite membranes having lower swelling percentages. However, excessive introduction of filler material can result in the membrane becoming stiffer and more fragile. Fuel cell testing revealed that the 10 wt. % composite membrane was the best performing, with it being closest in polarisation behaviour to recast Nafion. 

Saccà et al. [152] continued their work by studying the characteristics of Nafion-Titanium oxide membrane for PEMFCs operating at medium temperatures. Introduction of 3 wt. % of titania powder increased the water uptake from 20% for recast Nafion to 29%. The composite membrane outclassed commercial Nafion at all fuel cell operating temperatures (80, 90, 110 and 130 °C). At 110 °C (and 0.56 V), maximum power densities were 0.514 W cm^−2^ and 0.354 W cm^−2^ for the composite and commercial membrane respectively. As well as the better polarisation performance, the cell resistance of the composite membrane decreased with temperature up until 110 °C, where it starts to increase (0.106 Ω cm^−2^). This is in comparison to the commercial membrane whose resistance begins to increase after 100 °C (0.088 Ω cm^−2^). Interestingly, experiments with steam reforming fuel (with 10 ppm CO, 20% CO_2_, 75% H_2_ and 1% CH_4_) at 110 °C showed similar OCV values compared to pure hydrogen. Morever, the maximum current density decreased from 1300 mA cm^−2^ for pure hydrogen to 800 mA cm^−2^ for the synthetic fuel.

Amjadi et al. [153] also studied the influence of titanium oxide as a filler in Nafion composites, with two types of composites prepared via different methods, a solution casted and an in-situ sol-gel synthesis. EDX mapping across the composite membrane revealed that the sol-gel composite had a better dispersion of particles, which ultimately led to improved properties. One example is water uptake, where both composites had improved uptake capabilities compares to recast Nafion. However, due to the agglomeration and reduced uniformity in the casted composite, a decrease in surface area of the filler reduced the achieved water uptake. The introduction of filler led to a drop in proton conductivity, which was explained by the disruption of proton pathways in the membrane. Fuel cell testing at 110 °C showed that the composite membrane was able to reach a maximum current density of nearly 600 mA cm^−2^, compared to just over 200 mA cm^−2^ for Nafion 117. 

**Figure 4:**
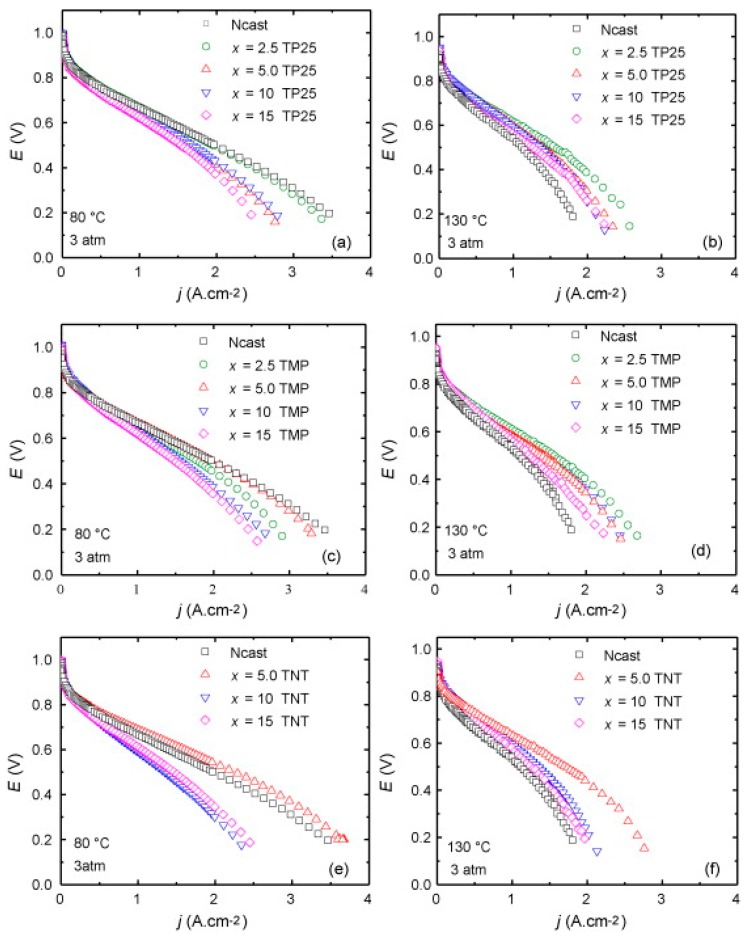
Polarization (***I–V)*** curves for the (**a**,**b**) TP25, (**c**,**d**) TMP, and (**e**,**f**) titanium oxide nanotube (TNT) composites measured at 80 and 130 °C; reproduced with permission from [154].

Furthermore, Matos et al. [154] studied the influence of particle shape (spherical nanoparticles, high surface area mesoporous particles, and nanotubes) of titania for the application of PEMFCs operating at temperatures up to 130 °C. Water uptake tests revealed that any addition of spherical or high surface area (HSA) titania led to a decrease in water uptake compared the recast Nafion, with greater decrease at higher filler loading. However, the water uptake for the titania nanotube composite membrane increased, reaching a maximum of nearly 60% at 15% loading, compared to 42% for recast Nafion. The authors state that this is because of the “nanotubular” structure in which water molecules being able to exist inside the nanotube. Single cell tests (Figure 4) were performed at 80 and 130 °C. Nafion outperformed the composite membranes at 80 °C, however, the membrane degraded significantly once the temperature increased. All three composite membranes displayed a smaller amount of decrease in polarisation at 130 °C in comparison to recast Nafion. However, increasing filler loading in all three prospective filler materials (nanoparticle, mesoporous particles and nanotubes) led to a decrease in polarisation, particularly in the ohmic region, which the authors explain due to the decreasing proton conductivity with greater filler loading.

Zhengbang et al. [155] synthesised titanium oxide nanowires as a filler material in Nafion for PEMFCs operating at a higher temperature in addition to reinforcing the mechanical properties of the membrane. Addition of the nanowires led to a subsequent drop in water uptake and swelling, with increasing loading leading to increased reductions. The reduced swelling would help maintain mechanical integrity at higher operating temperatures. Fuel cell testing at 90 °C showed that the composite membrane experienced a smaller drop in polarisation when the humidity was reduced, in comparison to Nafion where the change in polarisation was much greater. Humidity stress tests revealed that the composite membrane had less stress (which becomes smaller with increased loading) than the recast Nafion, which experienced a high level of humidity related stress indicating lower lifetime. 

**Figure 5:**
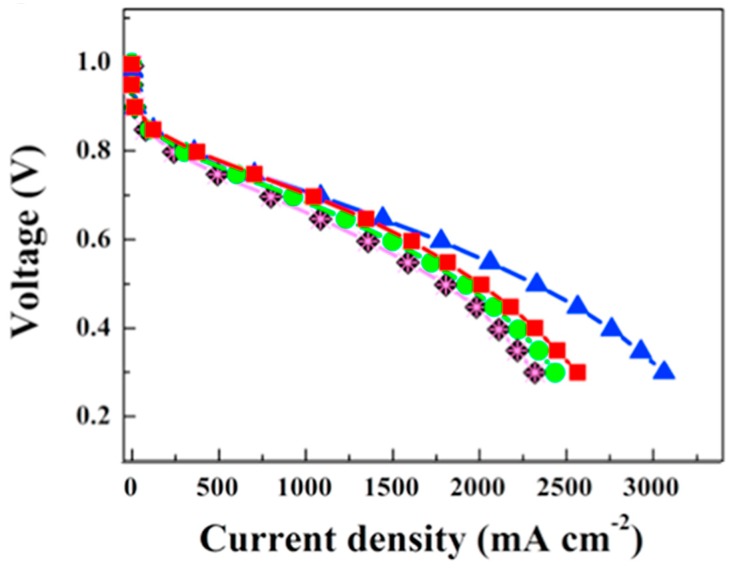
Polarization plots of Nafion 212 (black), recast Nafion (pink), Nafion TNT 10% (blue), Nafion TNT 20% (red), Nafion TNT 50% (green) at 80 °C and 100% RH; reproduced with permission from [156].

Ketpang et al. [156] further developed their idea of tubular inorganic fillers by studying the effect of titanium oxide nanotubes as a filler. The composite membranes had a higher water uptake compared to recast Nafion, with recast Nafion achieving 21.8%, Nafion-TiNT-10 33.7%, Nafion-TiNT-20 31.3% and the Nafion composite with 50% titanium oxide nanotubes achieving a water uptake of 29.6%. In addition, FT-IR analysis after drying the membranes at 110 °C revealed that the composite membranes still had water (from electrostatic interaction) through peaks that corresponded to -OH stretching (3455 cm^−^^1^) and –HOH- (1625 cm^−^^1^) bending vibration. Proton conductivity measurements at 80 °C and 100% RH confirmed that the filler improved the proton conductivity compared to recast Nafion (97 mS cm^−^^1^). The highest proton conductivity measurement was achieved by the composite with 10% filler (155 mS cm^−^^1^), with the 20% (142 mS cm^−^^1^) and 50% (121 mS cm^−^^1^) having slightly decreased conductivity. The composite membranes also outperformed the pristine Nafion at variable RH. Fuel cell experiments at 80 °C and 100% RH (Figure 5) show that the composite membranes perform much better than the recast membrane, with current densities at 0.6 V of 1777, 1609, 1498 and 1357 mA cm^−2^ for the composite membranes with filler of 10, 20, and 50% titanium oxide nanotube (TNT) content and recast Nafion, respectively.

The OCV also ranged from 0.97 to 1.03 V, indicating low crossover. Similar to the zirconium oxide nanotube, the titanium oxide nanotube composite displayed greater current densities at lower voltages. A 100 h stability test at 0.5 V, 80 °C and 18% RH showed that the composite membrane’s maximum power density decreased from 470 to 442 mW cm^−2^, whereas Nafion 212 only managed to produce a maximum power density of 55 mW cm^−2^ which degraded to 22 mW cm^−2^ after 100 h, an impressive difference in performance.

Jun et al. [157] then fabricated a Nafion composite with functionalised titanium oxide nanotubes and 3-mercaptopropyl-tri-methoxysilane (MPTMS) was used to functionalise the inorganic filler, to further improve proton conductivity. Nanotubes are a promising filler due to their high surface area and internal space, in addition to providing mechanical strength. In addition, the water uptake was greater, with 27.2 to 23.7%, for functionalised nanotubes to functionalised nanoparticles, respectively. Proton conductivity measurements at 120 °C and varying relative humidities show that the functionalised titania nanotubes exhibited greater conductivities than recast Nafion, at all humidities, with the deviation being greater at lower RH.

A Nafion composite comprising of porous zirconium oxide nanotubes were fabricated by Ketpang et al. for the purpose of high temperature PEMFCs [158]. The tubular structure of the filler was used to improve water transport, which should result in improved water uptake and proton conductivity. The performance of these composite membranes was tested at 80 °C at varying relative humidities of 100, 50 and 18%. It was found that the addition of the filler resulted in improved power densities at 0.6 V, implying that the filler lowers the ohmic resistance. In addition, the composite membrane revealed greater current densities at low voltages (0.3 V), this was explained due to the more efficient back diffusion from the cathode to the anode, mitigating flooding. A further 200 h durability test of the membrane (with 1.5 wt. % of filler) at the same operating conditions (80 °C and 18% RH) displayed a small decrease in OCV, from 0.99 to 0.92 V after 200 h. The authors have shown that use of a porous nanotube morphology can improve water transport and have potential advantages in low relative humidity application. 

Research into composite membranes extended beyond of the use of Nafion to other proton-conducting polymers. Marani et al. decided to combine sulphonated poly(ether ether ketone) (SPEEK) with titania nanosheets (an alternate material structure) for the application in PEMFCs operating at temperatures of 140 °C [159]. The authors studied the effect of treating the composite membranes with either water or with acid prior to use in addition to the effect of inorganic filler loading. It was found that acid treated membranes (with the lower filler loading of 1.67%) had the greatest proton conductivity in comparison to pristine SPEEK, with values of 4.14 × 10^−2^ Scm^−^^1^ at 140 °C and at 100% relative humidity to 1.76 × 10^−2^ Scm^−^^1^, respectively. This is because the acid washing displaced the tetrabutylammonium (TBA+), which was used to create the stable suspension of Titania nanosheets. However, acid treated membranes with higher loading displayed a porous structure and extreme swelling indicating chemical instability and high degradation rate.

Devrim et al. [160] fabricated a composite membrane with titanium oxide and sulphonated polysulfone as the polymer matrix. The degree of sulphonation of the polymer was varied and higher levels of sulphonation led to a higher water uptake, with a sulphonation degree of 15% providing a water uptake of 7%, compared to 33% for a sulphonation degree of 40%. Adding titanium oxide to the sulphonated polymer (40% sulphonation degree) resulted in a drop in water uptake, to 29%. The authors explain that this is because the introduction of the filler reduces the membranes’ free volume and ability to swell sufficiently. Proton conductivity values increased with increasing levels of sulphonation and temperature, with the composite of 40% sulphonated polysulfone/titanium oxide producing a conductivity of 0.098 S cm^−^^1^. Single cell tests at varying operating temperatures from 60 to 85 °C reveal that the pristine sulphonated polysulfone undergoes excessive swelling above 70 °C, leading to lower power output. The composite membrane outperforms the pristine reference membrane as the filler provides mechanical reinforcement to the membrane, preventing excessive swelling and deformation. The sulphonated polysulfone membrane produced a maximum power density of 0.16 W cm^−2^ at 85 °C, compared to 0.24 W cm^−2^ for the composite membrane.

Sambandan et al. synthesised silica and functionalised sulphonated silica composite membranes with SPEEK as polymer of choice [161]. Water uptake results show that the composite membrane had lower water uptakes compared to SPEEK. Fuel cell testing at 80 °C and 75%, in addition to 50% RH show that the composite membranes, particularly those with functionalised filler have polarisation curves similar to that of recast Nafion. Proton conductivity results for the functionalised composite membrane were 0.05 S cm^−^^1^ and 0.02 S^−^^1^ cm with the same operational parameters to the fuel cell, respectively. Therese at al. [162] prepared a SPEEK/PAI (poly amide imide) membrane with sulphonated silica filler. The PAI was added to the SPEEK to improve the mechanical strength and chemical resistance at higher operating temperatures. The composite membrane produced a proton conductivity of 8.12 × 10^−2^ S cm^−1^ at 90 °C. The idea of combining more than one polymer for composite membranes in an interesting one as instead of trying to choose one optimum polymer to work with, several can be blended.

Sahin et al. [163] produced a SPEEK cerium phosphate composite membrane to improve fuel cell performance and to increase oxidative stability. Fenton testing revealed that the composite membrane lost 10% in weight over 80 h, whereas the SPEEK membrane was completely destroyed. Proton conductivity also increased with filler content until 10% loading, where it begins to decrease.

Carbone et al. [164] fabricated a SPEEK composite membrane with amino-functionalised silica filler for elevated temperature operation in PEMFCs. Two types of SPEEK were synthesised, with 35 and 52% degree of sulphonation. The addition of the functionalised filler did not change the water uptake or swelling (at 100 °C) of the 35% sulphonated SPEEK. However, the 52% SPEEK water uptake and swelling dropped significantly with the addition of filler (from 400% to 120% water uptake and from 4 to 1.5 degree of swelling ratio). The authors explained that this is due to the strong sulphonic-aminic groups. Fuel cell testing at 120 °C showed that the composite membrane with 52% degree of sulphonation and with 20 wt. % of filler produced a peak power density of 246 mW cm^−2^ (around 400 mA cm^−2^) compared to 179 mW cm^−2^ (around 320 mA cm-2) for 52% SPEEK without filler. 

The same authors decided to continue this line of work and studied the effects of a zeolite filler (H-BETA) inside a SPEEK matrix for medium temperature fuel cells [165]. The introduction of zeolite reduced IEC of the SPEEK membrane (around 50% degree of sulphonation) from 1.55 to 1.47 (5% filler), 1.4 (10% filler) and 1.31 meq g^−^^1^ (15% filler). At 80 °C, the pristine SPEEK outperforms the three composite membranes but at 120 °C all three composite membranes outperform the SPEEK reference membrane. The composite membrane also had a higher OCV than the reference SPEEK. The authors explain this as the zeolite providing necessary mechanical reinforcement as well as retaining water in the membrane that would otherwise be removed at elevated temperatures.

Moreover, Ozdemir et al. investigated the addition of different inorganic fillers (silicon dioxide, titanium dioxide and zirconium phosphate) to PBI for high temperature PEMFCs [166]. The properties that the authors were looking for included improved acid uptake and greater acid retention (lower levels of leaching). All three prospective fillers led to decreased acid leaching, from pristine PBI lost 85.2% of its doped acid compared to SiO2/PBI at 81.5%, TiO_2_/PBI at 77.4% and ZrP/PBI at 75.9%. Also, SiO2/PBI and ZrP/PBI displayed improved proton conductivity values compared to pristine PBI. Both membranes produced their highest conductivity at 180 °C, with 0.113 and 0.200 S cm^−^^1^, respectively. However, TiO_2_/PBI displayed proton conductivities lower than pristine PBI. This was explained due to the non-uniform dispersion of filler (agglomeration) within the Nafion, which was observed on the SEM images. All four membranes conductivities increased with increasing temperature (140, 165 and 180 °C).

Lee et al. fabricated a PBI composite with sulfophenylated titanium oxide nanoparticles for fuel cells operating at elevated temperatures [167]. As expected, the introduction of the filler material improved acid retention and proton conductivity. The composite membrane produced a peak power output of 621 mW cm^−2^, whereas pristine PBI produced 471 mW cm^−2^, at 150 °C. One thing to note was that the membranes were very thin for composites, with film thicknesses of around 15 µm before acid doping and 22 µm after and therefore hydrogen crossover tests would be interesting to perform to understand the difference in crossover between pristine and composite membranes. 

Ooi et al. investigated improving the acid retention and oxidative stability of PBI membranes operating at increased temperatures [168]. This was achieved by preparing a composite membrane which composed of partially fluorinated PBI and a filler of cesium hydrogen sulfate-silicotungstic acid (CsHSO_4_–H_4_SiW_12_O_40_, CHS-WSiA). The synthesised composite exhibited greater acid retention rates, which was attributed to the fluorinated PBI and the filler material. This retention was examined in a fuel cell 24 h stability test, where a voltage of 0.614 V at a constant current of 0.2 A cm^−2^ was produced with no drop. A longer test would be interesting to validate the durability of the membrane.

Devrim et al. [169] prepared a silica polybenzimidazole (PBI) composite membrane for high temperature PEMFCs. The silica nanoparticles improved the acid retention and the proton conductivity. Proton conductivity results measured at 140, 165 and 180 °C revealed that the composite membrane had greater conductivity than pristine PBI, 0.0675 to 0.0600, 0.0866 to 0.0765, 0.1027 to 0.0944 Scm^−^^1^ for PBI/SiO_2_ respectively at the three temperatures. The addition of silica also reduced the degree of acid leaching from 41.5 (for pristine PBI) to 36.3% due to increased covalent bonding between the inorganic filler and acid. Single cell testing was also performed at the three temperatures previously stated, under hydrogen and air at 1 atmosphere. At 140 °C the pristine PBI outperformed the composite but at the two higher temperatures the composite membrane produced a greater maximum power density. The best performance was from the composite membrane at 165 °C, producing a maximum power density of 0.24 to 0.2 Wcm^−2^ for the pristine PBI, at 0.6 V. The authors have shown the novelty of using inorganic filler to retain acid in PBI for high temperature PEMFC applications.

Plackett et al. [170] tested laponite clay as a filler in PBI for high temperature fuel cells. Two sets of fillers were prepared, by functionalising the clay with an imidazole group and another with quaternary ammonium group. Water uptake results showed that no difference was made when the organic filler was introduced in the PBI matrix, but the composite membranes did experience less acid swelling. The composite membranes achieved an OCV of 1.02 V (at room temperature, 0.96 V at 125 °C and 0.91 V at 200 °C), which implies low or almost non-existent hydrogen crossover, which was confirmed in permeability tests. 

Aili et al. [171] doped silica with phosphotungstic acid for use as a filler in phosphoric acid doped polybenzimidazole for high temperature PEMFCs. This composite had a lower swelling rate due to its lower uptake of phosphoric acid. Durability testing at 200 °C revealed that the composite membrane had a decay rate of 27 μV h^−^^1^, whereas the membrane without the filler decayed at a rate of 129 μV h^−^^1^.

Other inorganic materials are used as they have the potential to improve the durability of the membrane. Rodgers et al. [172] used platinum nanoparticles as a filler to remove radicals formed during fuel cell operation and therefore reduce degradation. Membranes with 0, 10, 30, and 50 mol % of platinum were prepared, and their performance was evaluated in a 100 h fuel cell test at 90 °C and 100% RH. The highest degradation (through fluoride emission) was observed for the 10 mol % platinum composite. The authors explain that this is because of the low distribution and density of the platinum particles throughout the membrane. 

**Figure 6:**
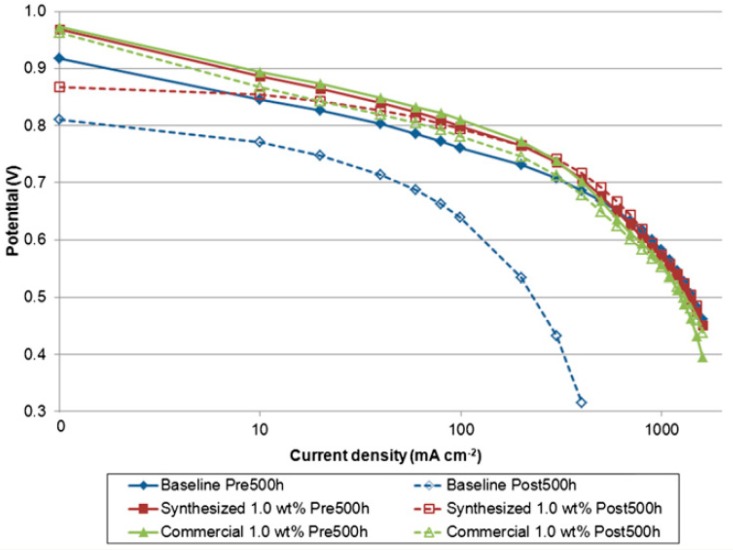
Pre- and post 500 h test performance curves; reproduced with permission from [173].

Pearman et al. [173] studied the influence of cerium oxide as a radical scavenger in PEMFCs. Two forms of cerium oxide were used as fillers within a PFSA polymer structure, a synthesised version with 2–5 nm sizing, and a commercial version with 20–150 nm. The addition of cerium oxide resulted in a 50% reduction in OCV decay rate (from a 94 h test), from 0.9 mV hr^−^^1^ for pristine Nafion to an average of around 0.4 mV hr^−^^1^. However, the weight percentage of cerium oxide seemed to make no difference in the decay rate. Electron microscopy images show that less platinum particles were present in the composite membrane in comparison to the recast. A 500 h OCV hold test with pre and post-test polarisation curves, depicted in Figure 6, demonstrated that the composite membranes had a much smaller deviation in polarisation compared to the baseline Nafion membrane. The authors followed this work up by studying the proton conductivity of the composite membranes [174]. Unfortunately, the composite membranes did not perform as well as the baseline Nafion, with long term conductivity testing resulting in a continuous decrease in conductivity (80 °C, 70% RH for 4 days). The authors discovered that this is due to the excess acidity of the PFSA, humidification and gas flow reducing the cerium oxide into (III) which then binds to the sulphonic groups, inhibiting proton conduction. This was confirmed by reprotonation via sulphuric acid and the proton conductivity went back to its original value.

Lee et al. [175] prepared cerium oxide impregnated sulfonated poly(arylene ether sulfone) (SPES, 50% degree of sulphonation) membranes aiming for improved fuel cell durability. The addition of cerium oxide led to a drop in water uptake, IEC and proton conductivity. With increasing loading resulting in lower water uptake, IEC and proton conductivity. However, the ex-situ Fenton reagent tests was performed to study the oxidative stability of the composite membranes. Introduction of the cerium oxide led to a decrease in degradation of the membrane. In addition, single cell accelerated OCV hold testing (90 °C, 30% RH, 0.5 bar) showed that the composite membrane with 2% cerium oxide was stable for up to 2200 h, compared to 670 h for the pristine SPES.

Elakkiya et al. decided to enhance the proton conductivity of composite membranes by using sulphonated TiO_2_ coated in polyaniline within a SPES polymer matrix [176]. Water uptake and proton conductivity improved with the addition of the filler however, no in-situ testing was performed. It would be interesting to see what effect the polyaniline has on fuel cell performance, and if the sulphonated filler improves performance at elevated temperatures/reduced humilities. 

Lee et al. [177] synthesized sulphonated silicon dioxide within SPAEK. As expected, the addition of the filler improved fuel cell performance (at 60 °C and at both 100 and 70% RH) but the functionalised filler also outperformed the composite with non-functionalised silicon dioxide. This is due to the sulphonic groups retaining more water and allowing sufficient proton transport.

### 3.2. Carbon Nanomaterial Fillers

In recent year carbon nanomaterials have become the go-to filler, particularly graphene oxide due to its abundance of oxygen containing functional groups [178]. 

These oxygen-containing functional groups attract water molecules and are able to retain higher levels of water in comparison to pristine Nafion. In addition, graphene oxide’s flat structure means that these functional groups are easily accessible. The inclusion of filler materials can also improve the mechanical strength of the composite membrane.

Kumar et al. [179] prepared a GO/Nafion membrane for PEMFCs operation. Addition of GO in 2, 4, and 6% loading to recast Nafion led to a subsequent increase in water up from 21.1 to 27.9, 37.2 and 36.1% respectively. Additionally, IEC changed from 0.891 to 1.21, 1.38 and 1.26 meq g^−^^1^ respectively. The authors argued that there is an optimum quantity of filler and any addition would result in increased membrane stiffness and subsequently reduced water uptake. Fuel cell tests at 100 and 25% RH show that the 4% GO composite membrane outperformed the reference recast Nafion by nearly 4 times (212 mW cm^−2^ to 56 mW cm^−2^).

Sahu et al. [180] instead functionalised graphene with sulfonic acid groups inside a Nafion matrix for low relative humidity operation. This is interesting as the use of graphene oxide as a filler is due to its abundant oxygen containing functional groups, which make it more hydrophilic. This is in comparison to graphene, which is hydrophobic and hard to disperse in water, however the sulfonation procedure would have reduced the hydrophobicity of the graphene filler. This is shown in the water uptake and IEC tests. Recast Nafion has a water uptake of 20.1%, the addition of graphene slightly increases it to 21.4%. However, the introduction of sulphonated graphene, in 0.5, 1 and 1.5% loading results in improved water uptakes of 24.5, 27.3 and 29.2% respectively. The IEC values are: 0.88, 0.89, 0.92, 0.96 and 0.95 meq g^−^^1^ respectively. A similar trend was also observed with the proton conductivity, with the 1% sulphonated graphene having the best performance, which is also hinted at by it having the highest IEC. Fuel cell testing at 70 °C and 20% RH revealed that the composite membrane with sulphonated graphene (1%) produced a maximum power density of 300 mW cm^−2^, whereas recast Nafion and Nafion-graphene (1%) produced peak power densities of 220 mW and 246 mW cm^−2^ respectively.

Lee et al. [181] prepared Nafion/GO and a novel Pt on graphene/Nafion composite membranes for low humidity PEMFCs. The idea behind using platinum on graphene as a filler is to use platinum as a reaction site to produce water and “self-humidify” the membrane. Water uptake experiments showed that the GO composite membrane outperformed the pristine Nafion sample. In comparison, the Pt/Graphene filler led to a drop in water uptake. The authors explained that this is because of the less hydrophilic nature of platinum as well as the GO being reduced to graphene in the synthesis step. However, the Pt/Graphene membrane had a greater proton conductivity compared to the other two membranes, which was explained via the electronic tunnel effect. The GO composite had a lower proton conductivity due to the filler impeding the ionic pathways, but this issue was resolved when the loading was greater than 3%, resulting in an increase in proton conductivity. The GO/Nafion membrane was tested at 80 °C and under a range of RH. At 40% RH, the peak power densities of the membranes with different GO loadings were all around 0.5-0.6 W cm^−2^. On the other hand, the Pt/Graphene membrane gave disappointing current output under anhydrous conditions, with peak current densities of 0.27, 0.36 and 0.14 A cm^−2^ for 0.5, 3 and 4% loading respectively. 

The authors followed up this work with designing a composite membrane with platinum on graphene in addition to silicon dioxide to improve the “self-humidifying” capabilities of the membrane by using the silica to retain the water produced by the platinum-graphene [182]. The water uptake and proton conductivity of these novel membranes increased with increasing silica content. Maximum water uptake of 30% was achieved with 3% Pt-G and 3% silica content. Fuel cell experiments showed that the addition of silica improved the polarisation curve. However, performance dropped with too much silica at low RH, which the authors explain is possibly due to the filler blocking the ionic pathways. Filler optimisation was concluded by the authors, as increases in Pt-G loading also resulted in a drop in performance.

Yang et al. [183] fabricated a composite membrane with platinum deposited on titania, which is then incorporated with graphene oxide into a Nafion polymer matrix. The composite membranes displayed a better IEC than recast Nafion, with increased until 20% GO is reached, where the IEC began to decrease beyond that. The proton conductivity followed a similar trend to the IEC, which also decrease past 20% GO loading. Fuel cell testing with varying levels of RH showed that the Pt-TiO_2_ improved the fuel cell performance, however the authors noted that this was still not sufficient at zero RH. Adding the GO led to an even greater improvement in cell polarisation. Nafion/0.8Pt–TiO_2_/0.2GO generated a current density of 0.54 A cm^−2^ at 0.6 V at 0% RH, which compared to Nafion/Pt–TiO_2_ that produced a current density of 0.01 A cm^−2^ at the same RH. Furthermore, the introduction of GO not only improved the current density generation, but also helped alleviate significant OCV loss when the humidity was lowered.

**Figure 7:**
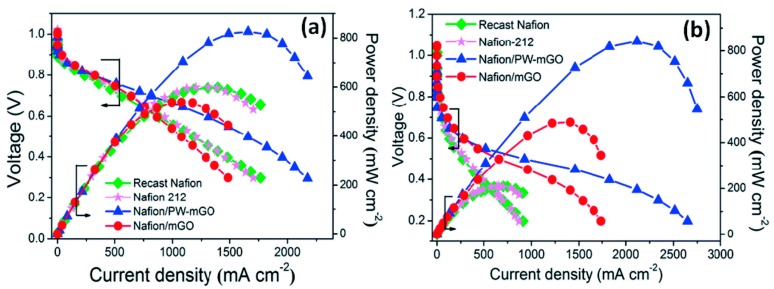
Polarization and power density plots of Nafion-212, recast Nafion, Nafion/mGO and Nafion/PW-mGO operating (**a**) under 100% RH at 80 °C and (**b**) under 20% RH at 80 °C. One weight percent filler content was used in composite membranes, and catalyst loading in the anode and cathode was kept 0.5 mg cm^−2^; reproduced with permission from [184].

Kim et al. [184] fabricated a GO/Nafion composite where the GO is modified with phosphotungstic acid (H_3_[PW_12_O_40_]·29H_2_O) to aid water uptake and proton conduction at low relative humidity PEMFC operation. Fuel cell testing at 80 °C and 20% RH showed that the composite membrane with modified GO produced a maximum power density of 841 mW cm^−2^, which is a great improvement in comparison to non-acid doped Nafion/GO which generated 488 mW cm^−2^, and 208 mW cm^−2^ for recast Nafion. Polarization curves are shown in Figure 7.

Maiti et al. [185] synthesised a composite Nafion membrane comprising of graphene oxide and an ionic liquid (dihydrogen phosphate functionalised imidazolium) for high temperature PEMFCs. TGA shows that the ionic liquid is stable up to 230 °C, which is more than enough for high temperature operation. The composite membranes displayed greater proton conductivity compared to Nafion 117 throughout the entire temperature range tested (70–110 °C). This improved performance was carried through to the single cell test where the composite membrane generated higher current densities in comparison to the Nafion 117 MEA (at 110 °C and dry conditions).

Branco et al. [186] investigated the performance of multilayer membranes for IT-PEFC applications. Multilayer membranes with two external Nafion outer layers and an inner layer of graphene oxide and another with sulphonated polyindene were fabricated with solution casting and hot-pressing methods. The solution casting protocol involves heating the first Nafion layer at 100 °C for two hours to remove the solvents in the Nafion dispersion, followed by the addition of the graphene oxide solution/sulphonated polyindene (in deionized water) and another two hours at the same temperature. Lastly, the final Nafion layer was added and the multilayer membrane was heat treated for one hour at 120 °C. The increased temperature is to anneal the polymer. Multilayer membranes that were casted displayed better performance and proton conductivity than the hot-pressed multilayer membranes. This was explained by the casted membranes having better interface interaction compared to the hot-pressed membranes, which suffered delamination. Multilayer membranes with sulphonated polyindene showed higher performance than Nafion at 120 °C.

Ibrahim et al. [187] studied the behaviour of GO composite membranes fabricated via solution casting with different thicknesses at intermediate operating temperatures. The composite membranes had improved mechanical strength and a higher water uptake in comparison to pristine Nafion. In-situ fuel cell testing of the membranes as MEAs revealed that the 30 μm composite membrane at 100 and 120 °C outperformed the 50 μm Nafion membrane at 80 °C. This is most likely due to the reduction in thickness and the GO filler retaining more water, hence reducing the drop in proton conductivity.

Kumar et al. [188] sulphonated GO and incorporated it into the polymer matrix of SPEEK. The SGO improved the water of SPEEK from 57.58% to 60%. In addition, the composite membrane outperformed SPEEK at temperatures from 30 °C to 80 °C (at 100% RH) and at 80 °C (with varying RH from 30 to 50%). Fuel cell testing at 80 °C, 30% RH humidified hydrogen and dry oxygen showed that the composite membrane produced a maximum power density of 378 mW cm^−2^, a large increase in comparison to SPEEK which produced 250 mW cm^−2^.

Sulphonated carbon nanotubes were used as a filler within a SPEEK matrix to offset the effect of high levels of sulphonation compromising the durability of the membrane [189]. The composite membrane had better proton conductivity and fuel cell performance compared to its pristine counterpart. A point of consideration is that the filler was functionalised to prevent the disruption of the proton transport channels, which is something that should be considered when incorporating a filler.

Uregen et al. [190] fabricated a graphene oxide/polybenzimidazole membrane for the operation at high temperatures. The introduction of graphene oxide improved the proton conduction in comparison to pristine PBI as well as reducing the quantity of acid leaching (from 85% for pristine PBI, to 70% for the composite membrane). Fuel cell testing at 165 °C and with dry hydrogen and air revealed that the pristine PBI and GO composite membrane had maximum power densities of 0.31 and 0.38 W cm^−2^ respectively. However, the authors noted that there could potentially be degradation of the GO functional groups at operating temperatures above 165 °C. A 500 h durability test showed that the performance loss of the composite membrane was lower, at 3.8% in comparison to 8.3% for the PBI membrane. This could be due to the reduced hydrogen crossover and acid leaching.

Xue et al. [191] decided to functionalise their graphite oxide, once again in a PBI polymer matrix for high temperature PEMFCs. Isocyanate functional groups were modified onto graphite oxide to improve the dispersion in water and organic media. This resulted in greater proton conductivity and less swelling. A similar study but with the GO sulphonated was studied by Xu et al. [192]. The proton conductivity of the membranes was increased from 0.023 S cm^−^^1^ for pristine PBI to 0.027 S cm^−^^1^ for GO/PBI and 0.052 S cm^−^^1^ for SGO/PBI. The respective activation energies for proton conduction fell from 16.1 kJ mol^−^^1^ to 11.4 kJ mol^−^^1^ to 9.3 kJ mol^−^^1^ respectively. Fuel cell testing at 175 °C and under anhydrous conditions with hydrogen and oxygen showed that the addition of GO or SGO result in an increase in maximum power density, from 0.22 for PBI, to 0.38 for GO/PBI and 0.6 W cm^−2^ for SGO/PBI. The same trend was observed under air. This work was followed by the same authors studying the same filler and polymer but this time functionalised the GO with an ionic liquid (1-(3-Aminopropyl)-3-methylimidazolium groups) [193]. The composite membrane had a higher proton conductivity in comparison to the reference PBI membrane. Fuel cell tests at 175 °C with dry inlet fuel showed that the addition of the ionic liquid improved peak power densities from 0.26 W cm^−2^ for PBI to 0.32 W cm^−2^ for the composite. The authors stated that this is due to the improved proton conduction within the composite membrane.Abouzari-Lotf et al. [194] designed a composite membrane for high temperature fuel cells by combining PBI that has been functionalized with 2,6-Pyridine with phosphonated grapene oxide. The use of the filler was in order to reduce the extent of acid leaching and to increase long term stability as increasing acid content can mechanically compromise the polymer. The addition of 1.5% phosphonated graphene oxide significantly increased the proton conductivity from 19.6 × 10^−3^ S cm^−1^ for pyridine PBI to 76.4 × 10^−3^ S cm^−1^ at 140 °C.

**Figure 8:**
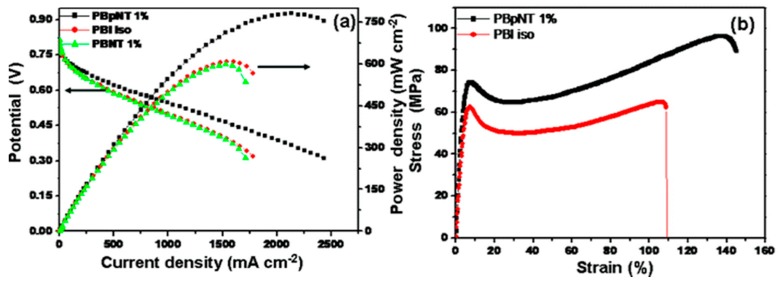
(**a**) Polarization plots of PBI iso, PBpNT, and PBNT composite membranes measured at 140 °C by passing dry H_2_ and O_2_ at a flow rate of 0.2 slpm. The cells were conditioned at 0.6 V for 30 min. (**b**) Stress−strain curve for the pristine PBI and PBpNT composite membrane; reproduced with permission from [195].

Kannan et al. [195] presented a composite PBI membrane consisting of phosphonic acid functionalised multi-walled carbon nanotubes as the filler material. Proton conductivity tests revealed that the composite membrane achieved 0.11 S cm^−^^1^, whereas the pristine PBI produced a conductivity of 0.07 S cm^−^^1^. Figure 8 show fuel cell testing at 140 °C with dry inlet feeds showed that the composite membrane (1% filler loading) outperformed both the pristine PBI membrane and an additional composite that contained non-functionalised nanotubes (peak power densities of 780, 600 and 590 mW cm^−2^ respectively). In addition, the mechanical stability was also improved due to the architecture of the carbon nanotubes, achieving a higher yield strength.

A further more detailed study involved various characterisation techniques and an investigation on variable loading [196]. Thermal analysis revealed that the membranes are stable up to 250 °C, and from 250 °C to 400 °C only lose 10% mass. The composite membranes all produced a proton conduction greater than pristine PBI throughout the experimental temperature range. The phosphoric acid uptake was similar for both the pristine membrane and the composite of different loadings. However, the activation energy dropped from 40.9 kJ mol^−^^1^ for PBI to 25.1 kJ mol^−^^1^ for the composite membrane with 1% functionalised CNTs. The composite membranes experience a smaller drop in activation compared to the reference membrane and the authors explained this as the catalysts (platinum) having a higher exchange current density on CNT than carbon. Additionally, the composite membrane produced higher current densities without a sudden drop due to concentration limitations, being able to reach nearly 3000 mA cm^−2^, in comparison to nearly 2000 mA cm^−2^ for pristine PBI.

Yang et al. [197] used GO as a filler functionalised with triazole groups in order to aid dispersion and to improve proton conduction within PBI for high temperature fuel cells. SEM imaging revealed that the modified GO composite had a much better dispersion in comparison to the non-modified GO composite which had the presence of agglomerates. This improved homogeneity led to an increase in proton conductivity as well as an improvement in its mechanical properties. Fuel cell testing at 160 and 180 °C showed that the composite membrane outperformed the PBI reference membrane at the same acid doping level, with maximum power densities of 537 to 506 mW cm^−2^ respectively.

Cao et al. [198] fabricated a graphene oxide poly (ethylene oxide) (PEO) composite membrane for the purpose of PEMFCs operation. The conductivity of the composite membrane increased from 0.086 S cm to 0.134 S cm with increasing temperature (from 25 °C to 60 °C). However, the authors explained that increasing the temperature above that results in the membrane softening. The composite membrane produced a maximum power density of 53 mW cm^−2^ at 60 °C with full humidity.

Lee et al. [199] prepared a SPAES composite with GO grafted onto sulfonated poly(arylene thioether sulfone) as the filler. This was done due to the inherent lower proton conductivity of hydrocarbon-based polymers in comparison to PFSA. This membrane exhibited improved mechanical strength and oxidative resistance, as well as better proton conductivity in comparison to pristine SPAES. Fuel cell testing to understand the performance of this composite as an MEA would be very interesting, and whether grafting the GO makes a difference in performance. 

Dai et al. [200] developed novel composite membranes consisting of carbon dots of different sizes and with varying levels of hydrophilicity within a matrix of polyvinylpyrrolidone (PVP) and polyethersulfone (PES). AFM and TEM characterization showed that carbon dots with a size of 2–5 nm showed no aggregation and good uniformity. Single cell tests at 150 °C and under anhydrous conditions revealed that the composite membrane had a higher peak power density in comparison to pristine PES-PVP, 166 to 113 mW cm^−2^, respectively. The idea of altering the hydrophilicity of the filler material is an interesting technique to improving the performance of the composite membrane.Ahmed et al. prepared a chitosan membrane with sulphonated multiwall carbon nanotube filler [201]. As chitosan has a lower proton conductivity than Nafion there is a greater need for using fillers to improve its proton conductivity. The mechanical strength and proton conductivity increased but water uptake decreased, and the authors explain that this is due to the decrease in -NH_2_ functional groups. It would be interesting to see how these membranes perform in a fuel cell in comparison to pristine chitosan and Nafion. 

### 3.3. Acids and Ionic Liquids Fillers

Ionic liquids have been extensively used in fuel cells operating at higher temperatures due to their high thermal degradation temperature. For example, Choi et al. fabricated two types of composite membranes doped with phosphotungstic acid, one with 1100EW Nafion and the other with 750EW [202]. At 120 °C, both the composite membranes performed better than the reference Nafion MEA, achieving a voltage of 0.51 and 0.55 V (1100EW and 750EW) at 400 mA cm^−2^, compared to 0.47 V for the reference Nafion. In addition, the ohmic resistance was smaller than that of the reference Nafion, at 0.32, 0.21 and 0.13 Ω cm^−2^ for Nafion, 1100EW composite and 750EW composite respectively.

Lee et al. designed a composite membrane of a sulphonated polymer doped with fluorohydrogenate ionic liquid [203]. The ionic liquid was used due to their high thermal stability as the application was geared towards intermediate temperature operation with dry conditions. Single cell testing at 130 °C revealed an OCV of 1 V for 5 h. In addition, the ionic conductivity of the prepared composite membrane increased with temperature, from 11.3 mS cm^−2^ at 25 °C to 34.7 mS cm^−2^ at 130 °C.

Ramani et al. [204] introduced heteropolyacids into Nafion for PEMFCs operating at higher temperatures and reduced relative humidity. Additives studied included phosphotungstic (PTA), silicotungstic (STA), phosphomolybdic acid (PMA) and silicomolybdic acid (SMA). Water uptake results revealed that there is no significant different between recast Nafion and the Nafion/PTA composite membrane at a range of relative humidities. Single cell tests at 120 °C and 35% RH of Nafion/PTA, Nafion/STA and Nafion/SMA showed relative performance, with Nafion/PTA and Nafion/STA reaching a maximum current density of around 800 mA cm^−2^. The same authors prepared a composite membrane of Nafion with a heteropolyacid (HPA), phosphotungstic acid (PTA) [205]. The MEA was “stabilized” via high temperature heat treatment (200 °C at 30 atm). In order to allow the membrane to not disintegrate and to prevent the HPA from dissolving, the MEA was ion exchanged in caesium carbonate, swapping the protons for much larger caesium ions. TGA experiments showed that this stabilized membrane degraded at higher temperatures in comparison to its proton exchanged counterpart. Fuel cell testing at 120 °C and at 35% RH showed that both the stabilized and reference membrane have similar polarisation behaviour. However, the specific area resistance was lower for the stabilized membrane and the authors explained that this is because of the lower contact resistance from the high temperature heat treatment. The work was followed by looking into the effect of extent of ion exchange. Composite membranes with 2, 1, and 0 protons left after substitution were prepared [206]. Weight loss measurements to assess the stability of PTA in Nafion were performed, with increasing proton substitution leading to less weight loss after protonation. Pristine PTA had a weight loss of around 27%, which decreased to less than 5% for the PTA modified to have its protons removed. Water uptake experiments interestingly showed that there is no difference between pristine PTA composite and the substituted protons. In addition, membranes were also ion exchanged using different cations, but this led to no change. The authors proposed that they think that any improvement in proton conductivity in the fabricated membranes will be exclusively because of the Grotthus mechanism, with negligible contribution via the vehicular mechanism.

Another avenue to reduce leaching of the HOA was to use metal dioxides as a support, in a similar fashion to carbon for the electrocatalysts [207]. TGA analysis showed that the addition of the PTA and metal dioxide (silica in this case), increased the membrane decomposition temperature from 270 to 305 °C, and this is because of the silica partially immobilising the side chains of the Nafion. FT-IR before and after protonation treatment revealed that the PTA did not wash out of the composite membranes that were prepared via sol-gel technique. In-situ resistance measurements at 120 °C and 35 °C show that the composite membranes (with PTA supported on silica) have an area specific resistance of 0.16 Ω cm^−2^ in comparison to 0.19 Ω cm^−2^ for Nafion.

**Figure 9:**
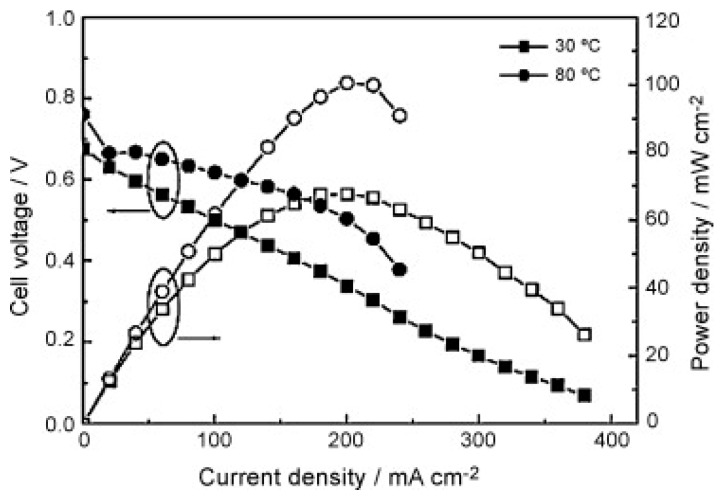
Polarization curves of a H_2_/O_2_ fuel cell using a SPI-1.51(50) composite membrane without humidification at gas utilization ratios of 30% for H_2_ and 15% for O_2_ (■,□) operation at 30 °C, (●,○) operation at 80 °C; reproduced with permission from [208].

Lee et al. [208] prepared a membrane with a protic ionic liquid diethylmethylammonium trifluoromethanesulfonate ([dema] [TfO]), within a sulphonated polyimides (SPI) structure for anhydrous PEMFCs. 300 °C was estimated as the composite membrane’s thermal decomposition temperature, which is much higher than its intended PEMFC operating temperature. Fuel cell operation at 80 °C and under dry conditions, illustrated in Figure 9, revealed that the composite membrane produces a maximum power density of 100 mW cm^−2^ at a current density of 240 mA cm^−2^. What is interesting is that at 30 °C, a maximum power density of 68 mA cm^−2^ at 300 mA cm^−2^, showing some promise of a room temperature, anhydrous PEFC.

Yi et al. [209] fabricated a SPEEK ionic liquid composite membrane, based on an imidazolium ionic liquid, for increased temperature and anhydrous fuel cell operation. Two composite membranes were prepared, one with 1-butyl-3-methylimidazolium tetrafluoroborate (BuMeImBF4) and the other with 1-decyl-3-methylimidazolium tetrafluoroborate (DeMeImBF4). In addition, the degree of sulphonation of the SPEEK was chosen to be 67%. Proton conductivity measurements of SPEEK/BuMeImBF4 showed that the conductivity increased with increasing temperature, reaching a maximum of 8.4 × 10^−3^ S cm^−^^1^ at 170 °. The authors explain that the increase is due to the reduction in viscosity of the ionic liquid enhances its mobility. Thermal analysis via TGA showed that the composite membrane’s sulphonic groups degrades at 340 °C, which is much greater than that of 250 °C of pristine SPEEK. Leaching tests were performed to understand how much of the ionic liquid would be retained in the composite membrane. Proton conductivity tests of the composite membranes before and after immersion in water showed that the proton conductivity of SPEEK/BuMeImBF4 was undetectable after 1 h of immersion. SPEEK/DeMeImBF4 fared better, being detectable after 2 h of immersion but eventually the conductivity also became undetectable.

Yasuda et al. [210] synthesised sulfonate polyimide (SPI)/ionic liquid composite membranes with altered polymer structures, to study the effect of the positions of the sulfonic groups, ultimately for anhydrous application. Composite membranes with random and block polymers were made and characterised to understand their behaviour. The degradation onset temperature for all the membranes were 250 °C and above, this means that they are suitable for operating in higher temperature fuel cells. The authors stated that the distribution of the ionic groups and the flexibility of the sulfonic groups are important determinants in ion conduction. Fuel cell experiments at 120 °C and with no humidity showed that the random chain SPI and homopolymer SPI produced maximum power densities of 100 and 70 mW cm^−2^ respectively.

Malik et al. [211] prepared a SPEEK/ethylene glycol/ionic liquid composite membrane for high temperature application. The ethylene glycol was added to use as a crosslinker to help alleviate the quantity of leaching. The composite membranes had a high thermal stability, with the sulphonic groups beginning to degrade at 240 °C. The composite membranes had a lower leaching weight loss in comparison to the non-crosslinked membranes, however the proton conductivity of the crosslinked membranes was lower. The authors explained that some of the sulphonic groups where used in the crosslinking resulting in lower conductivity.

From the above analysis of the literature, it can be seen that membrane fillers are very versatile, in terms of chemical structure, size, dimensions, etc. Numerous different characteristics to adapt to specific application whether that is operating at high temperature, low relative humidity, increasing mechanical strength, preventing acid leaching, increasing proton conductivity, or producing self-humidifying membranes. On top of that they can also be functionalised to either boost these characteristics or provide a secondary functionality. The abovementioned studies indicate promising performance for composite membranes; however, highlight the need for further research to improve the lifetime and durability of these membranes. Table 3. summarises hydrogen PEM performance of composite polymer electrolyte membrane described in this review.

Membranes with fillers that were functionalised (most commonly with sulphonic groups) displayed a better performance in terms of proton conductivity and cell polarisation at elevated temperatures. This is attributed to the water retaining capabilities of these functional groups. Different fillers demonstrated different performances, and this is because of their chemical structure in addition to their physical structure (nanoparticle, flat, nanotubes). Therefore, when selecting a filler material not only should the material itself and possible functionalising be considered, but also the shape of the filler itself. Another point of consideration is the polymer that the filler will be embedded in. As we have shown, composite membranes were made using different polymers such as Nafion, SPEEK and SPAES. However, only Nafion meets industry standards regarding lifetime and durability. This implies that composite membranes should use a Nafion matrix in addition to Nafion ionomer in the GDE. Overall, membrane performance has to be looked at from a variety of experiments, such as; cell polarisation and power, long term durability and ex-situ tests to name a few. A membrane that performs well in-situ might degrade quickly during thermal/humidity cycling and be unsuitable.

Another point of consideration is the interaction between the composite membrane and the catalyst layer. Conventional Nafion membranes use gas diffusion electrodes that consist of Nafion ionomer binder. However the addition of a filler material could potentially affect this interaction between the membrane and catalyst layer, for example the filler is added to only improve the membrane performance but if some of the filler is dispersed closer to the edges of the membrane then this could interfere with the anode and cathode functions (hydrophilic fillers situated close to the cathode could cause flooding more easily). Also, the ionomer and membrane might not consist of the same material, further complicating this interaction, which would be exacerbated during manufacturing of MEAs with composite membranes and binders of different materials. To the authors knowledge, the use of composite ionomers in the catalyst layer is not studied and requires further research.

Composite membranes are tested for their performance in-situ (fuel cell testing) and ex-situ (proton conductivity, water uptake etc.), however their durability during fuel cell testing is an area of research not fully explored. In order to be competitive with conventional Nafion membranes, the composite membrane must not only be able to perform better but also perform adequately over long periods. Composite membranes are developed aiming for harsher operating conditions (higher temperature and lower humidity) and therefore their durability must be investigated and demonstrated in-situ. It is also important to study the change in the membrane degradation mechanisms due to the presence of fillers, for instance; how does the filler affect the membrane mechanical properties due to the humidity cycling and what is the impact of the filler on the catalyst stability or dissolution into the membrane.

## 4. Composite Membranes for Electrolysers

**Figure 10:**
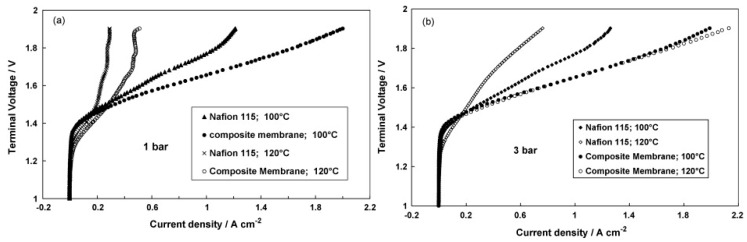
Comparison of voltage and current density of Nafion 115 and composite Nafion-SiO_2_ membrane at 100 and 120 °C and at 1 bar abs (**a**) and 3 bar abs (**b**); reproduced with permission from [213].

Composite membranes with metal oxides as fillers (SiO_2_, TiO_2_, or WO_2_) showed promising properties for high temperature operation of PEM water electrolysers allowing achieving high performance with respect to a commercial membrane. Baglio et al. [212] and Antonucci et al. [213] focused their work on Nafion-TiO_2_ and Nafion-SiO_2_ respectively, to allow efficient operation at high temperature, above 100 °C. Both works claimed that the high temperature operating conditions were allowed by the better water retention and more uniform distribution of water across the composite membrane due to the presence of inorganic hygroscopic fillers inside the polymeric matrix. This resulted in reduced ohmic resistance and therefore better electrolyser performance [214]. The performance of composite membranes was better than that of Nafion membrane under high temperature and high pressure so the application of this technology is very promising especially when high electrical efficiency is required. As evidence of this result, Figure 10 illustrates characteristics curve of cell equipped with commercial Nafion and composite Nafion-SiO_2_ membranes at high temperature and pressure.

These alternative composite membranes also showed a decrease of the cross-over of the gases through the membrane. However, a slight decay of performance was observed during the experiment; thus, a further amelioration of membrane is necessary to improve the stability and lifetime.

Another way to produce electrolyte membranes with high conductivity and durability for water electrolysers is using perfluorosulfonic acid with shorter and non-branched pendant side-chain with higher crystallinity than longer side-chain perfluorosulfonic acid. Aricò et al. [215] used the Aquivion short side chain perfluorosolfonic membrane using Nafion 111 for comparison. Authors claimed that although those membranes showed high conductivity, mechanical stability and dimensional properties, they are not appropriate for water electrolysis application. To reinforce those membranes, organic fillers can be included in the Aquivion matrix. Boaretti et al. [216] included SPEEK reinforcement which led to an improvement in the mechanical strength but resulting in low proton conductivity. Another approach to reinforce proton exchange membrane is to physically separate the properties of mechanical strength and proton transport embedding a porous nanofibre web into the matrix. Aquivion membranes reinforced with electrospun polysulfone (PSU) fibre webs were prepared by Giancola et al. [217]. The fibrous reinforcement strongly enhanced the mechanical strength and also reduced hydrogen crossover. However, the addition of the reinforcing fibre in membranes had little effect on the cell electrochemical performance: the cell voltage at 2 A cm^−2^ was 1.760 V which is slightly higher than the performance obtained with a non-reinforced membrane (1.758 V). Therefore, increased mechanical and dimensional stability and reduced hydrogen crossover of the composite membrane are promising properties for electrolysis application but with little effect on performance.

Ion-Ebrasu et al. [218] produced a composite membrane by spray coating graphene on commercial PEM material. They exploited the properties of this material to enhance the efficiency of PEM electrolysers and reducing costs by achieving high surface area to volume ratio, good mechanical and thermal properties. The composite membrane showed an improved behaviour in term of thermal and electrochemical characterization when compared to pristine commercial membrane: the interaction of graphene with fluorinated membrane led to an increased conductivity and a better water adsorption. In spite of all this benefits, further experimental work has to be carried out to investigate the behaviour of these graphene-modified membranes under current voltage measurements.

Linkous et al. [219] evaluated different types of engineering polymers and identified a few options that could withstand the conditions found in PEMWEs. Among them, polybenzimidazoles (PBI), poly(ether ether ketones) (PEEK), poly(ether sulfones) (PES) and sulfonated polyphenyl quinoxaline (SPPQ), were selected to be used for PEM electrolysis. In particular, SPEEK polymer is considered to have high strength and it is an easy membrane forming material. High degree of sulfonation enables high proton conductivity. In fact, Linkous et al. observed that high degree of sulfonation (65%) led to a higher proton conductivity that exceeded Nafion by 29%. However, these alternative membranes showed low durability and low current densities compared to standard Nafion membranes and tent to swell excessively or even dissolve at elevated temperature. An alternative would be to reinforce the SPEEK membrane with other polymer structures and/or fillers [220]. Song. et al. [221] prepared a composite membrane including tungstophosphoric acid(TPA) to increase proton conductivity and CeO_2_ (Cs) to improve the durability of the membrane into the SPEEK matrix. The composite membrane showed better mechanical and electrochemical properties than Nafion 117 membrane: proton conductivity, tensile strength, and elongation were enhanced. However, the cell voltages of the MEA using Nafion 117 and SPEEK-Cs/TPA membrane were 1.91V and 1.82 V at 1 A cm^−2^ operating at 80 °C under atmospheric pressure; thus, they may replace Nafion 117 due to their mechanical characteristic, electrochemical properties once performance becomes comparable with that of Nafion117.

It can be noted that despite the promising characteristics of composite membranes, little research has been conducted into using them in PEMWEs. It is important to note that in addition to Nafion, and similar to what is reported for fuel cells, other polymers have been explored for PEMWE application. The polymers include: SPEEK, SPSU and PBI which have been employed with varying levels of performance and lifetime achieved. Again, these polymers can also be modified and made into composite membranes for PEMWEs for achieving both higher temperature and pressure operation. Table 4 summarizes the properties and the pros and the cons of composite electrolyte membranes described in this section compared to those of the commercial membrane. In addition, Table 5 summarizes their best power output obtained:

As illustrated in the tables above, despite having better water retention, composite membranes are not yet a suitable alternative to the commercial one in terms of performance and durability. Several efforts should be made to achieve performance industrially reasonable.

## 5. Conclusions

This review analysed several composite membranes developed in recent years for the use in PEM technologies to overcome the drawbacks of the commercial perfluorosulfonated membranes. Composite fluorinated, with organic and inorganic fillers, and non-fluorinated membranes have been scrutinized for DMFC, hydrogen PEMFC, and PEMWE.

All materials reported in this paper show promising characteristics and results, so it is not possible to indicate which one is the best. It can be noted that papers reporting high performance are dealing with the incorporation of fillers into the Nafion matrix, suggesting that Nafion cannot be completely replaced yet. Beyond the use of organic fillers like PBI and PANI, whose effects are evident only at high methanol concentration, carbon and inorganic fillers are the most promising materials. Low weight percentage of graphene oxide contributes to sensibly lower methanol crossover leading to better performance also a low methanol concentration. Moreover, GO composite membrane extends the operating temperature range for hydrogen PEMFC due to the fact that GO retains more water, so it decreases the loss in proton conductivity. Among all the composite membranes described in this review paper, inorganic fillers are the most versatile materials: their good thermal stability, improved water uptake and reduced methanol absorbance, provided high power density for DMFC and PEMFC but also allow high temperature and pressure operating conditions for electrolysis. Ionic liquids can be potentially used at intermediate temperatures once performance increases. Despite these positive results, durability tests are necessary to understand the real capacity of those fillers. Other materials like SPEEK and PVA are used to completely substitute Nafion. They seem to be a promising alternative to obtain high performance membranes. Research activities on their potentialities are still ongoing.

## Figures and Tables

**Table 1:** Summary of DMFC composite membrane properties.

Membrane	Preparation Method	Pros	Cons
**Nafion/PTFE**	Impregnation	Low methanol permeability	Decreased conductivity
**Nafion/PVA**	Casting	Low methanol permeability Easily manipulation with small thickness	Lower proton conductivity
**Nafion/PBI**	Screen printing	Reduced methanol permeability	High impedance
**Nafion/Polypyrrole**	Electrodeposition-In situ polymerization	Low methanol permeability	Decreased proton conductivity Increased resistances
**Nafion/Polyaniline**	In situ-polymerization	Decreased methanol permeability Increased selectivity	Decreased conductivity
**Nafion/SPAEK**	Casting	Low methanol permeability Higher proton conductivity	Easily breakable
**Nafion/SPEEK**	Casting	Decreased methanol permeability Reasonable thermal properties	Reduced proton conductivity
**Nafion/Metal oxides (SiO_2_-TiO_2_)**	Casting	Increased proton conductivity	Accelerated degradation Difficult homogeneity
**Nafion/Montmorillonite**	Casting	Methanol crossover decreased	Slight proton conductivity decrease
**Nafion/Zeolites (Analcime-Mordenite)**	Spray	Methanol crossover decreased Slight increased proton conductivity	Low tensile strength
**Nafion/Graphene oxide**	Casting	Methanol crossover decreased High thermal and mechanical stability	Decreased proton conductivity
**SPEEK**	Casting	Low methanol crossover	Poor mechanical stability
**SPAES/Laponite**	Casting	Low methanol crossover Enhanced tensile strength	Low proton conductivity
**PVA/Montmorillonite**	Casting	Low methanol crossover Cheap High proton conductivity	Filler content should be well controlled Specific operating condition and specific stack material should be used
**PVDF/silica-Zirconium**	Impregnation	High tensile strength Low methanol crossover	Poor proton conductivity

**Table 2:** Summary of DMFC best performance using composite membranes.

Membrane	Type of DMFC	Voltage (V)	Current Density (A.cm^−2^)	Power Density (mW cm^−2^)	Temperature (°C)	Methanol Concentration (M)
Nafion/silica [79]	Active	0.3	0.2	60.0	75	5
Nafion/TiO2 [83]	Active	0.3	0.214	64.2	80	1
Nafion/SiO2 [83]	Active	0.3	0.204	62.9	80	1
Nafion/sulfonated montmorillonite [87]	Active	0.2	0.336	67.2	40	2
Nafion/GO [98]	Active	0.31	0.46	141.0	70	1
Nafion/SGO [99]	Active	0.40	0.1	43.0	-	1
Sandwich Nafion/GO [100]	Passive	0.17	0.15	25.0	-	5
Nafion/mordenite [95]	Active	0.18	0.06	10.8	70	4
Nafion/analcime [95]	Active	0.18	0.04	7.2	70	4
Nafion/mordenite/GO [96]	Active	0.23	0.12	27.5	70	1
Nafion/polypirrole [64]	Active	0.30	0.15	45.0	60	
Nafion/polyaniline [65]	Active	0.23	0.3	70.0	60	6
Nafion/PVA [53]	Active	0.26	0.5	130.0	70	1
Nafion/PBI [58]	Active	0.36	0.06	21.7	60	2
Nafion/PTFE [47]	Active	0.25	0.35	87.5	70	2
Nafion/PTFE/zirconium phosphate [50]	Active	0.20	0.3	60.0	80	2
Nafion/SPAEK [72]	Active	0.38	0.3	114.0	80	2
Nafion/SPEEK [73]	Active	0.18	0.15	27.0	80	2
SPEEK [112]	Active	0.20	0.076	15.2	60	1
SPEEK/MMT [112]	Active	0.20	0.1	20.0	60	1
SPEEK/GO [116]	Active	0.35	0.21	72.2	65	1
SPEEK/PBI [113]	Active	0.28	0.16	45.0	60	1
PVA/montmorillonite [129]	Active	0.29	0.023	6.8	25	2
PVDF/zirconium phosphate [131]	Active	0.54	0.060	32.3	60	1

**Table 3:** Summary of hydrogen PEMFC best performance using composite membranes.

Membrane	Power Density (mW cm^−2^)	Temperature (°C)	RH%
Nafion/Silica [144]	350	100	100
Nafion/Silica particles [139]	380	85	100
Nafion/Hafnium oxide [147]	336	100	-
Nafion/Titanium oxide [152]	514	110	-
Nafion/Titanium oxide nanotubes [156]	1020	80	-
Nafion/Zirconium oxide [141]	400	130	85
Nafion/Sulphonated zirconium oxide [142]	609	70	83
Nafion/mesoporous zirconium pshosphate [146]	353	70	18
Nafion/ zirconium pshosphate [145]	450	130	-
Nafion/GO [179]	212	100	25
Nafion/SGO [180]	300	70	20
Nafion/GO/TiO_2_ [183]	324		0
Nafion/GO/Phosphotungstic acid [184]	841	80	20
Nafion/Phosphotungstic acid [202]	220	120	-
PBI [169] PBI/SiO_2_ [169]	200240	165165	-
PBI/GO [190]	388	165	0
PBI/SGO [191]	600	175	0
SulfonatedPolysulfone [160]	160	85	-
SPolysulfone/titanium oxide [160]	240	85	-
SPEEK [164]	179	120	-
SPEEK/GO [188]	378	80	30
SPEEK/silica [164]	246	120	-
SPI/ionic liquid [210]	100	120	0
SPI/demaTfO [208]	100	80	0

**Table 4:** Summary of electrolyser composite membrane properties.

Membrane	Preparation Method	Pros	Cons
**Nafion/Metal oxide**	Casting	Better water retention	Improvement registered at temperature greater than 100 °C and high pressure- Poor stability
**Aquivion**	Not present	Better water retention	Acceptable performance at certain temperature range, low humidity and high pressure
**Aquivion reinforced with polysulfone**	Casting	Low hydrogen crossover- Good mechanical stability	Performance not so much higher than non-reinforced membrane
**SPEEK**	Casting	Higher proton conductivity	Low durability and low performance at elevated temperature
**SPEEK-TPA**	Casting	Better chemical and mechanical stability	Performance slightly lower

**Table 5:** Summary of PEM electrolyser best performance using composite membranes.

Membrane	Voltage (V)	Current Density (A cm^−2^)	Temperature (°C)	Pressure
Nafion/SiO_2_ [213]	1.9	2.1	120	3 bar
Nafion/TiO_2_ [212]	2	1.46	120	3 bar
SPEEK [219]	2.5	0.21	60	atmospheric
SPEEK/TPA/Ce [221]	1.82	1	80	atmospheric
Aquivion/PSU [217]	1.76	2	80	0.1 Mpa absolute
SPSf [222]	1.8	1.08	80	atmospheric
